# Evaluation of the Fruit Quality and Phytochemical Compounds in Peach and Nectarine Cultivars

**DOI:** 10.3390/plants12081618

**Published:** 2023-04-12

**Authors:** Raffaella Petruccelli, Alessandra Bonetti, Leonardo Ciaccheri, Francesca Ieri, Tommaso Ganino, Cecilia Faraloni

**Affiliations:** 1 Institute of BioEconomy, National Research Council (CNR-IBE), Via Madonna del Piano n. 10, Sesto Fiorentino, 50019 Florence, Italy; raffaella.petruccelli@ibe.cnr.it (R.P.);; 2 Research Institute on Terrestrial Ecosystems, National Research Council (CNR-IRET), Via Madonna del Piano n. 10, Sesto Fiorentino, 10, 50019 Florence, Italy; 3 Institute of Applied Physics ‘Nello Carrara’ (IFAC), Via Madonna del Piano n. 10, Sesto Fiorentino, 50019 Florence, Italy; 4 Institute of Biosciences and Bioresources, National Research Council (IBBR-CNR), Via Madonna del Piano n. 10, Sesto Fiorentino, 50019 Florence, Italy; 5 Department of Food and Drug, University of Parma, Parco Area delle Scienze 27/A, 43124 Parma, Italy

**Keywords:** *Prunus persica*, quality parameters, sugars, phytochemical compounds, antioxidant activity

## Abstract

Qualitative traits and chemical properties of 32 peach cultivars (yellow flesh and white flesh fruits) and 52 nectarine cultivars (yellow flesh and white flesh fruits) of different pomological characteristics is performed, and the correlation between cultivars and chemical characteristics is analyzed. Yellow nectarines have a higher variability in soluble solids concentration (SSC) and titratable acidity (TA) values. Evaluation of color parameters (a*, b*, L*) shows a significant interaction between pulp color (white vs. yellow) and types (peaches vs. nectarines) of fruit. The difference between yellow and white fruits is stronger in nectarines than in peaches. Sucrose is the main sugar detected in peach fruits, with a percentage content of 78.37% and 76.70% of the total sugar content in yellow and white peaches, respectively, and 78.29% and 78.12% in yellow and white nectarines, respectively. Variability is found among cultivars for the chemical compounds analyzed. The yellow flesh has higher amounts of total carotenoids and TPC, while white-flesh fruits present an average antioxidant value higher than yellow-flesh fruits. No significant correlation is found for polyphenol content and DPPH, while an interaction (*p* < 0.005) between neochlorogenic acid content and peaches and nectarines is evidenced, with a neochlorogenic acid content higher in nectarines than in peaches.

## 1. Introduction

Peaches (*Prunus persica* L. Batsch) and nectarines (*Prunus persica* L. Batsch var nectarina [Ait] maxim) are the third-most economically important fruit tree crops after apples (*Malus* spp.) and pears (*Pyrus* spp.) [1,2]. The production world of peaches and nectarines exceeds 24.5 million tons on an area of approximately 1.5 million hectares, and China, Italy, the USA, Spain, and Greece are the major peach producers [3,4]. The fruits are used mainly for fresh consumption, but to a lesser extent, they are also used as dried fruit or as an ingredient in processed products (juices, jams, yogurt, and liqueurs) [3].

From a commercial point of view, peaches are classified into five pomological groups: yellow-fleshed peaches, white-fleshed peaches, yellow-fleshed nectarines, white-fleshed nectarines, and percoche. Nectarines are essentially indistinguishable from peaches; nectarine fruits have a smooth skin because of the lack of trichomes and exhibit subtle variations in flavor and size compared to the fuzzy peach. Percoche are peaches with firm and non-melting flesh used for direct consumption or for processing [5,6]. Based on the texture and firmness of the fruit, peaches are classified with melting, non-melting, and stone-hard flesh; based on the adhesion of the pulp to the stone (endocarp), a further classification can be made: freestone, clingstone, or semi-freestone pits [7,8]. 

In addition, cultivars are also classified according to (i) flowering time (very early, early, intermediate, late, and very late), (ii) ripening time (extra-early, early, intermediate, late, and very late), and (iii) fruit utilization (direct or fresh consumption, or industrial processing). According to fruit acidity, peaches are further classified into high-acid and low-acid cultivars [3,8]. Other characters that contribute to the classification of this species are yield, quality (taste, aroma, texture, and flavor), sensory qualities (sweetness, acidity, and astringency), nutritional and health properties, and resistance to different stress conditions [6,8,9,10]. Peaches and nectarines have the highest number of new cultivars released every year by intensive breeding programs worldwide. This contributes to the expansion and diversification of the range of fruit types, with different quality characteristics (taste, aroma, texture, and flavor), sensory qualities (sweetness, acidity, and astringency), and nutritional and health properties that are beneficial to human health [11,12,13].

In particular, varietal differences have been reported for primary (sugars, organic acids, amino acids, and dietary fiber) and secondary metabolites (vitamins, terpenes, and phenolic compounds) [3,10,14]. For example, soluble sugars (sucrose, fructose, glucose, and sorbitol) are responsible for the sweetness, and organic acid (malic and citric acids) correlate with the sensory perception of sourness, while phenolic compounds inhibit brown rot, but they are also the responsible factors for fruit sensorial-organoleptic attributes (flavor, aroma, and color pigmentation) and for the response of plants to biotic and abiotic stresses [15,16]. In addition, these same compounds are also important for human health, and peaches, like other fruits for fresh consumption, are considered pro-health fruits [17,18]. Recently, peach was considered a ‘functional food’ because of the presence of highly nutritious compounds and low calorie content. Several studies have shown their antihypertensive, anticarcinogenic, hypoglycemic, and anti-neuro-degenerative in vitro actions [19,20,21,22,23]. The chemical composition of peach fruits is mainly influenced by the cultivar, but also by type of rootstock, climatic conditions (water and light availability, soil composition, stresses, etc.), agronomic practices, harvesting time, and post-harvest factors (storage conditions) [24,25,26,27,28].

The objective of our study was to determine the qualitative indices and chemical attributes of 32 peach cultivars (yellow flesh and white flesh fruits) and 52 nectarine cultivars (yellow flesh and white flesh fruits) to implement the knowledge of this worldwide consumed fruit with important functions for human health.

## 2. Results and Discussion

Table S1 shows the carpological and agronomic characteristics of the cultivars analyzed. Weights of fruits ranged from 218.58 g (Maria Silvia cv) to 148.1 g (Lizbeth cv) for yellow peaches and from 218.33 g (Greta cv) to 178.76 g (Maria Bianca cv) for white peaches. Yellow nectarines showed weights between 253.7 g (Orion cv) and 124.5 g (Maria Dorata cv), while white nectarines ranged between 128.7 g (Maria Linda cv) and 212.9 g (Maria Anna cv). Harvest maturity was predominantly between intermediate and late for all cultivars analyzed.

### 2.1. Quality Parameters

The values of soluble solids concentration (SSC), titratable acidity (TA), and pH determined on peach and nectarine fruit pulp are shown in Table 1 and Table 2. Soluble solids content is an important element of peach fruit quality, and it can be a suitable marker of ripeness. The value of SSC is strictly dependent on the concentration of sugars. In yellow peaches, SSC value, at commercial harvest, showed an average value of 12.1 °Brix, with a range between 13.86 °Brix (‘Rome Star’) and 9.38 °Brix (‘Vistarich’). White peaches had an average value of 11.6 °Brix; the lowest content was in ‘Rosa del West’ (9.53 °Brix) and the highest in ‘Greta’ (13.23 °Brix). Percoche showed values between 9.90 and 13.27 °Brix in ‘Babygold 7’ and ‘Cotogna del Poggio’, respectively (Table 1). Titratable acidity was in a range between 11.05 g malic acid L^−1^ for ‘Summer Rich’ and 7.11 g malic acid L^−1^ for ‘Elegant Lady’ and ‘Rome Star’. White peaches had lower acidity values than yellow peaches with an average value of 8.22 g malic acid L^−1^. ‘Tardivo Zulari’ cv. showed the highest index while ‘Michelini’ the lowest, 9.76 and 6.63 g malic acid L^−1^, respectively (Table 1). 

Yellow nectarines showed significant intercultivar variability in relation to SSC and titratable acidity values (Table 2). SSC values were between 16.3 °Brix (‘Sweet Lady’) and 9.01 °Brix (‘Licinia’), while TA indices ranged from 13.9 to 6.08 g malic acid L^−1^. The highest TA values were recorded in the ‘Independence’, ‘Maria Aurelia’, and ‘Red Jewel’ cultivars, which had a slightly sour taste (Table 2). White nectarines displayed SSC values ranging from 14.23 to 10.49 °Brix with a mean value of 12.3 °Brix; TA indices ranged from 15.15 (‘Silver Star’) to 6.95 g malic acid L^−1^ (‘Maria Linda’) (Table 2). 

**Figure 1:**
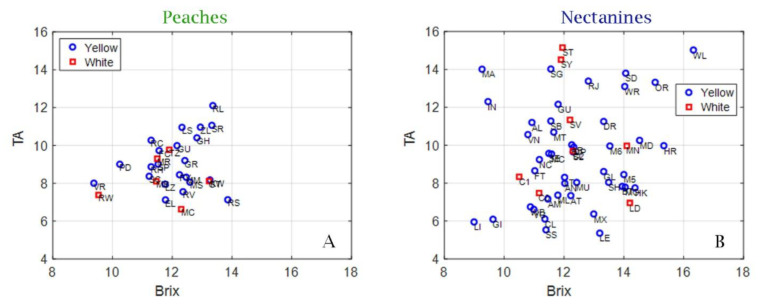
Scatter plots of °Brix and TA for 32 peach cultivars (yellow and white) (**A**) and 52 nectarine cultivars (yellow and white) (**B**).

From the analysis of the correlation between TA and SSC, it appears that these data showed a greater dispersion in nectarines than in peaches. This indicates that nectarines showed greater intervarietal variability than peaches. In nectarines, there is a greater variability in relation to SSC and titratable acidity values. However, both plots show no variability between yellow-flesh and white-flesh fruits in both nectarine and peach cultivars (Figure 1).

The SSC and TA values obtained in our study were similar to those found in the literature, both for peaches and/or nectarines and for flesh color, yellow and white. For SSC values, Tomas-Barberan et al. [28] reported ranges from 10.1% to 12% in peaches and 11.2% to 14.8% in nectarines; and Drogoudi et al. [29] reported ranges from 9.6% to 13.4% and 9.5% to 14.4% for peaches and nectarines, respectively. Gil et al. [24] reported TA values of 0.13–0.31% in white-fleshed peaches and 0.45–0.87% in yellow-fleshed peaches; nectarine values were 0.28–0.46 and 0.51–1.01 in white-flesh and yellow-flesh fruits, respectively. TA has been reported to range from 0.53–0.97% in yellow-flesh peaches, from 0.15–0.34% in white peaches, 0.31–0.47 and 0.66–1.16 in white- and yellow-flesh nectarines and from 0.53 to 086% in peaches and 0.63–0.93 in nectarines [29]. Reig et al. [30] and Baccichet et al. [31] obtained TA values ranging from 1.18 to 12.31 (g malic acid L^−1^). 

Regarding pH, values above 4.4 were recorded in the ‘Greta’ and ‘Maria Marta’ cultivars (yellow-fleshed peaches), while the ‘Maria Silvia’, ‘Suncrest’, ‘Red Valley’, and ‘Vistarich’ cultivars showed pH values slightly above 3.50 (Table 1). All white-fleshed cultivars showed pH values below 4.0, while the percoche showed values close to or above 4.0 (Table 1). TA was lower and pH was higher in the yellow-flesh peaches, while white flesh showed higher TA values and no difference for pH (Table 1). The pH value of yellow nectarines was between 4.73 and 3.05, and it was between 4.69 and 3.53 in white nectarines (Table 2). The average pH of yellow-fleshed fruits ranged from 3.81 to 3.92, while that of white-fleshed fruits was between 3.71 and 3.79. These values are normal for fruits of normal acidity (3). The pH and TA values of the fruit are related at this acidity. Peaches are classified as low acid or normal when the pH values are below 3.8 or above 4; while TA values ranged from 0.07 to 0.14% in low-acid cultivars and from 1.10 to 1.45% in acid cultivars [3]. 

The ripening index (RI = SS/TA) ranged from 1.65 (‘Elegant Lady’) to 1.11 (‘Red Coast’) in yellow-flesh peaches (Table 1) and from 0.66 (‘Maria Aurelia’) to 2.46 (‘Lady Erica’) in yellow-flesh nectarines (Table 2). White peaches (Table 1) and white nectarines (Table 2) had mean RI values of 1.44 and 1.27, respectively. RI is commonly considered an indicator of ripeness and an index for assessing the intensity of flavor of peaches and nectarines. It is the combination of sweetness and tartness that influences taste perception and consumer acceptance [29,32]. For each qualitative variable analyzed (SSC, TA, pH and RI), the results of our study revealed intercultivar variabilities. However, two-way ANOVA analysis showed no significantly different pairs (*p* < 0.05) among the samples analyzed with respect to type and color for the dependent variables SSC, pH, and RI, while a significant interaction emerged between total acidity and type (peaches vs. nectarines) (Table S2a–d).

Several authors propose to use qualitative parameters to define the quality of peaches: SSC was used as a sweetness indicator, TA as a sourness index, and the SSC/TA ratio as a flavor parameter [30,32,33,34,35]. Although these parameters are important indicators of the marketability of peaches, the preference of the consumer is linked to the cultivar; moreover, it has been observed that the choice and perception of the taste of the fruit depends on the relationship between the concentration of soluble solids and titratable acidity, rather than only on the value of SSC [24,30,34]. Crisosto and Crisosto [35] found that consumers preferred fruits with low acidity (<0.90%) and higher SSC content (>12.0%). Nevertheless, fruit qualitative parameters depend on several factors (environmental conditions, cultural practices, fruit position within tree canopy, and rootstock), and a wide range of variability was found for these parameters between cultivars and, in some cases, between plants of the same cultivar (plasticity). For this reason, ranges for quality the index were defined for both different types of peaches (yellow and white) and nectarines (yellow and white-flesh) and different cultivars [3,28,35,36,37]. 

The color parameters of peaches and nectarines (yellow and white) are reported in Table 1 and Table 2. In yellow peaches, the L* value ranged from 101.22 to 44.36; the higher one was found in ‘Symphonie’, whereas ‘Glohaven’ and ‘Zee Lady’ showed the lowest values, 44.36 and 44.76, respectively. In white peaches, the detected L* values ranged from 51.37 (‘Michelini’) to 40.02 (‘Maria Regina’) (Table 1). The range of means of the a* and b* values for the yellow cultivars examined was between 10.15 and −1.41 and between 32.64 and 6.48, respectively. In white-flesh peach cultivars, the a* values ranged from 0.87 to 5.44 and the b* from 29.93 to 12.82. (Table 1). In yellow-flesh nectarines, the mean value for the L* parameter was 53.21, and the highest L value was found in Spring Bright (97.38). The cultivar ‘Lady Star’ had the reddest color (high a* value) and ‘Orion’ the most intense yellow (high b* value). 

**Figure 2:**
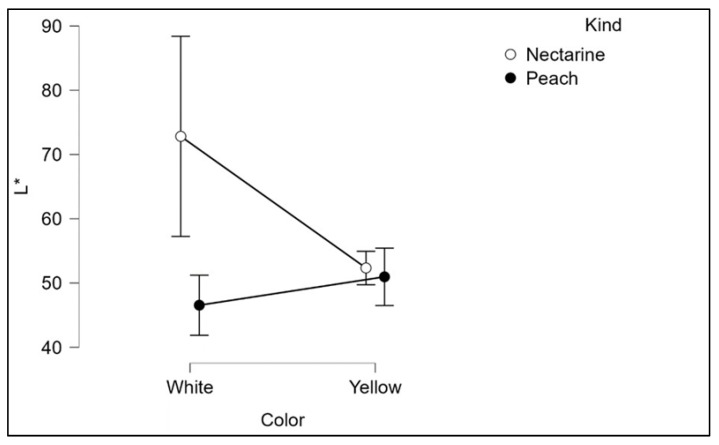
Scatter plots showing statistical interaction between color and type in peach and nectarine cultivars.

Two-way ANOVA showed that L* exhibits a significant interaction between pulp color (white vs. yellow) and types of fruit (peaches vs. nectarines). Figure 2 showed that the difference between the mean L values in white and yellow fruits is much stronger in nectarines than in peaches, with an opposite sign. 

Color is the characteristic commonly associated with fruit maturity, and fruit appearance is the first factor evaluated to define fruit maturity at harvest [37]. Color parameters, determined in the juice and puree of various peach and nectarine cultivars, have been reported by several authors [38,39]. Lavelli et al. [38] reported values of 0.90 (a*), 23.12 (b*), and 48.4 (L) for the puree of the Redhaven cultivar, and values of 9.62, 19.52, and 39.9 for a*, b*, and L, respectively, in Suncrest, whereas Versari et al. [39], in peach juices obtained from Redhaven cultivars, recorded values of 46 (L), −90 (a*), and 27 (b*), showing some differences from those found in our study.

The color of peach fruit is affected by the content of carotenoids, and their concentration and allocation vary among cultivars, as also between peel and flesh [40,41,42,43]. In addition, it correlates with some quality parameters of peaches and nectarines, and it is one of the major factors that the consumer uses to evaluate the quality of fruits [3,40,41].

### 2.2. Sugar Content

According to the literature [3,42,43,44], peach fruit contains different types of soluble sugars and sugar alcohols. Sucrose (non-reducing sugar) is the main sugar in peach pulp at maturity (40–80% of the total sugar content), followed by fructose and glucose (reducing sugar, 10–25% of the total sugar content) and sorbitol (<10%) [43]. The statistical analysis of carbohydrate concentration in all the peach and nectarine fruits analyzed is shown in Table 3.

In our study, sucrose was present with highest quantities (mean 67.22 g kg^−1^), followed by lower levels of fructose and glucose (8.06 and 8.79 g kg^−1^, respectively), confirming the results obtained by other authors [45,46,47,48]. Sugar alcohol sorbitol, a very important translocated sugar, showed a concentration between 0.70 and 4.44 g kg^−1^ with an average concentration of 1.90 g kg^−1^. The total amount of sugars ranged from 60.11 to 115.29 kg^−1^ FW, with an average concentration of 85.96 g kg^−1^ FW. 

Carbohydrate contents determined in 32 peach (yellow and white pulp) and 52 nectarine (yellow and white-flesh) cultivars are reported in Tables S3 and S4, respectively. Yellow-fleshed peaches Grenat, Lizbeth, and Symphonie cvs showed the higher contents of sucrose, with values greater than 75 g kg^−1^ FW; while the Summer Rich, Guglielmina, and Vistarich cultivars showed the lowest levels between 48.96 and 51.65 g kg^−1^. Sucrose content ranged from 61.05 (‘Tardivo Zuliani’) to 76.08 (‘Michelini’) g kg^−1^ in white peaches (Table S3). In the nectarine cultivars examined, sucrose values ranged from 84.89 (‘August Red’) to 52.28 (‘Gianna’, ‘Laura’, and ‘Dolce’) g kg^−1^ FW and from 66.48 (‘Caldesi 2000’) to 53.48 (‘Silver Star’) g kg^−1^ in white nectarines, respectively. In yellow peaches, glucose concentration varied from 12.49 to 4.14 g kg^−1^, with ‘Symphonie’, ‘Fayette’, and ‘Maria Marta’ presenting the higher values, while in the white peach cultivars, it varied from 12.09 (‘Maria Bianca’) to 7.29 g kg^−1^ (‘Tardivo Zuliani’) (Table S3). In yellow flesh nectarines, ‘Alma’, ‘Big Top’, ‘Fire Top’, and ‘Weinberger’ exhibited the highest concentrations of glucose and fructose (Table S4). In white nectarines, the higher fructose contents have been recorded in Caldesi 2000 and Maria Anna cvs, while ‘Maria Linda’ and ‘Silver Giant’ showed the lowest fructose contents of 5.61 and 5.93 g kg^−1^ FW, respectively (Table S4). Finally, sorbitol was determined in concentrations from 1.32 (‘Rome Star’) to 1.04 g kg^−1^ in yellow peaches and from 1.05 (‘Rosa del West’) to 0.70 g kg^−1^ (‘Maria Bianca’) in white peaches (Table S3). In the nectarine cultivars examined, sorbitol varied from 4.44 (‘Fire Top’) to 1.05 (‘Lady Erika’) g kg^−1^ FW and from 3.32 (‘Caldesi 2010’) to 1.12 (‘Maria Anna’) g kg^−1^ FW in yellow and white nectarines, respectively (Table S4). 

Table 4 summarizes the average content of sucrose, glucose, fructose, and sorbitol in yellow and white peaches and nectarines.

Sucrose, the dominant sugar, represented 78.37% and 76.70% of the total sugar content in yellow and white peaches, respectively. Nectarines are characterized by a high sucrose content, being at 78.29% and 78.12% in yellow and white fruits, respectively (Table 4). Glucose represented 9.38% and 9.18% of the total sugar content in yellow fruits of peach and nectarine, while the white fruits of peaches and nectarines exhibited values of 11.54% and 8.61%, respectively (Table 4). The concentrations of both total sugars and individual soluble sugars were comparable to the values reported in previous papers [49,50,51]. The ratio glucose/fructose ranged from 0.80 (white-flesh nectarines) to 1.08 (white-flesh peaches) and was significantly and positively correlated between them. The correlation coefficient was 0.648, with a 95% confidence interval ranging from 0.504 to 0.758. Specifically, Cirilli et al. [43] reported that, generally, fructose and glucose are present in equal amounts; the glucose/fructose ratio remains constant during fruit development and at maturity reaches values between 0.8 and 1.0. As suggested by several authors [48,52], fruits with low glucose/fructose ratios are preferable because fructose and glucose have a different degree of sweetness that affects fruit taste. 

**Figure 3:**
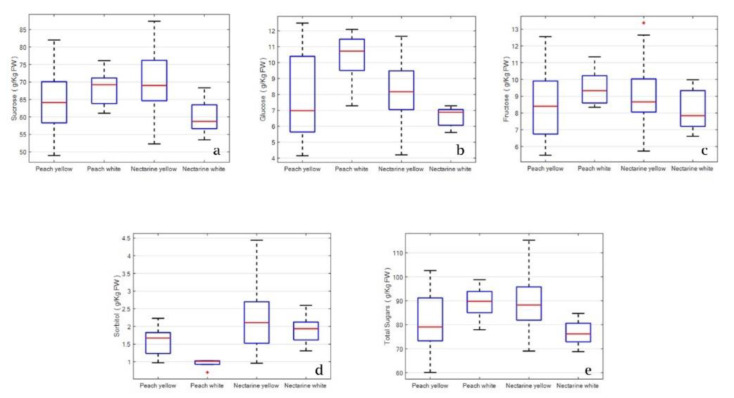
Box plots for sugars content of different types of peaches and nectarines fruits: (**a**) sucrose content; (**b**) glucose content; (**c**) fructose content; (**d**) sorbitol content; (**e**) total sugars. The central line displays the median, the bottom and top of the box are the first and third quartiles, respectively.

Sugars were analyzed by one-way and two-way ANOVA for kind (peach vs. nectarine) and color (white vs. yellow). In one-way analysis, each factor is considered separately, and no statistically significant differences in the concentration of sucrose, glucose, fructose, and total sugars were found (data not shown). The next step was to evaluate the sugar differences between types of fruit (peach vs. nectarine) and colors (white vs. yellow). Data were compared by two-way ANOVA analysis, considering, simultaneously, both factors and their interaction. The results are presented in Table 5 and Figure 3a–e.

Two-way ANOVA analysis showed a statistical significance (*p* < 0.005) between type of fruit (peaches and nectarines) and color (yellow and white) for all the samples analyzed. The main quantitative differences were observed for sucrose, glucose, sorbitol, and total sugars, while no factor was significant for fructose with two-way ANOVA (Table 5). Box plots show that sucrose was the highest in white fruits for peaches and in yellow fruits for nectarines. Nectarines also have a stronger difference between medians (Figure 3a). Type of fruit and fruit color interaction are highly significant for glucose concentrations (*p* ≤ 0.001) (Table 5). The box plot showed that glucose concentration is significantly higher in white fruits for peaches and in yellow fruits for nectarines. Peaches show a much stronger difference between medians. Glucose is particularly high in white peaches (Figure 3b). Fructose levels did not show any statistical differences (Table 5 and Figure 3c). Two-way analysis showed significant differences in type of fruit (*p* < 0.001) and fruit color (0.024). In particular, sorbitol content was particularly low in white-flesh peaches (Table 5 and Figure 3d). For total sugar, only interaction type (peaches vs. nectarine) and color (white vs. yellow) were highly significant (*p* < 0.002). This is due to the contribution of sucrose and glucose (the two main sugars). Indeed, the usual pattern observed in the box plot showed more sugar in white fruit for peaches and in yellow fruits for nectarines (Table 5 and Figure 3e).

Several studies have evaluated sugar composition in commercial cultivars, in local accessions, and in intra- and interspecific progenies [30,45,46,47,48]. Colarič and collaborators [46] investigated sugar profiles of 11 yellow-fleshed peaches, 2 white-fleshed peaches, and 6 yellow-fleshed nectarine cultivars. The authors observed a greater total sugar content in white peaches (84.97 g kg^−1^ FW) than in yellow peaches (74.09 g kg^−1^ FW) and in yellow nectarines (85.44 g kg^−1^ FW) than yellow peaches. Reig et al. [30] evaluated the total and individual sugar contents of 106 peaches, reporting statistically significant differences between the samples analyzed: higher values in yellow and white nectarines and lower values in yellow and white peaches, respectively. Wide variability in the total sugar content and individual carbohydrate profiles has been reported in a study that investigated the sugar composition of 94 accessions of peach and nectarine cultivars (local Spanish and worldwide cvs) [49]. 

Recently, Wanpeng et al. [50] showed no differences in the sucrose, glucose, fructose, and sorbitol determined in 18 commercial cultivars (honey peach, nectarine, and flat peach). The sucrose content varied from 69.29 (flat peach) to 44.79 (honey peach) mg·g^−1^ FW, while glucose ranged from 3.97 (nectarines) to 6.02 (honey peach) mg g^−1^ FW. Mrázová et al. [18], analyzing 34 peach cultivars (9 white-fleshed and 25 yellow-fleshed peach), observed that the average value of total sugars was about 13.6 g 100 g^−1^ FW in yellow fruits and 10.86 g 100 g^−1^ FW in white fruits.

Total carbohydrates and specific sugar profile have a pronounced influence on both the quality and flavor of fruit, influencing the degree of sweetness and caloric value. The level of sweetness is considered one of the main attributes used in the nutritional and sensory evaluation of fruit and is crucial in assessing consumer satisfaction with peach taste. In addition, increasing sugar content is the main objective in the selection of new cultivars with improved fruit quality and high consumer acceptance [9,43,51].

### 2.3. Determination of Total Carotenoids, Total Phenolic, and DPPH Assay

Peach and nectarine fruits are reservoirs of various biomolecules (carotenoids, polyphenols, and anthocyanins) that modulate physiological processes in the plant (protective chemicals against abiotic or biotic factors) [15,16] and influence the color and organoleptic characteristics of the fruits and exert beneficial effects on human health [3,21,23,47]. 

Carotenoids are the main pigments responsible for flesh colors, providing attractive yellow, orange, and golden colors, and their total content is higher in yellow-flesh peach cultivars than in white-flesh peach cultivars [3,40]. The contents of total carotenoids in commercial peach and nectarine varieties are shown in Table 6. In the extracts of white-flesh peaches, the total carotenoid concentrations ranged from 1.22 to 8.22 mg g^−1^ for ‘Tardivo Zuliani’ and ‘Maria Bianca’, respectively. The average value for this group was 3.93 mg g^−1^. The average content of total carotenoids in the evaluated yellow-flesh peaches was 6.98 mg g^−1^ (Table 6).

**Figure 4:**
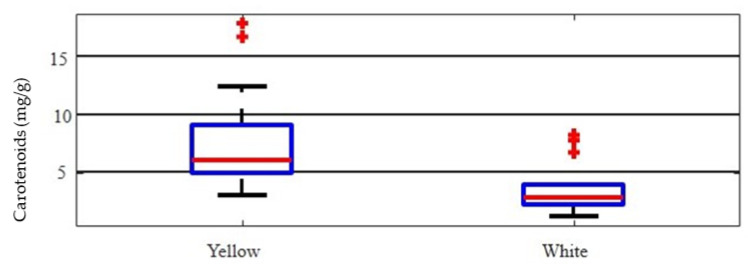
Box plots for total carotenoid contents (mg g^−1^) of yellow- and white-flesh fruits. The central line displays the median, the bottom and top of the box are the first and third quartiles, respectively.

In white-fleshed nectarines, the average concentration of total carotenoids was 3.43 mg g^−1^; the Maria Anna cultivar exhibited the highest value (6.71 mg g^−1^). The total carotenoid amount found in the extracts of yellow-flesh peaches was in a large range of 3.03–16.65 mg g^−1^. The cultivars with the highest content were ‘Maria Silvia’, ‘Rich Lady’, and ‘Vistarich’. Among the yellow-fleshed nectarines, Licinia and Vega cvs showed the highest contents, 12.38 and 16.65 mg g^−1^, respectively; in contrast, Guglielmina, Lizbeth, and Symphonie cvs recorded the lowest carotenoid contents (Table 6). In this study, the mean concentration of carotenoids, found in yellow-flesh types (peaches and nectarines), almost doubled that observed in the white-fleshed fruits (Figure 4), confirming results previously reported by other authors [24,40].

Gill et al. [24] reported carotenoid levels of 128.4–9.6 μg/100 g (yellow white nectarines), and 23.2–131.6 μg/100 g (white and yellow peaches). Vizzotto et al. [52] analyzed the carotenoid content of 19 peach genotypes. In this case, the accumulation of carotenoids in yellow-fleshed peaches was 35 times greater (2.80 mg/100 g) than in white fruits (0.08 mg/100 g). 

Recently, Mrázová et al. [18] reported that the content of total carotenoids of peaches was 0.33 and 2.18 mg/100 g in white and yellow fruits, respectively. The correlation between total and specific carotenoids (lutein, β-cryptoxanthin, α-carotene, and β-carotene) and flesh colors and/or color coordinates (L*, a*, b*) was observed by some authors. The highest content of carotene and carotenoids seems to be present in peaches with more intensely colored flesh [24,40,53].

Tourjee et al. [54] related colorimetric values with total carotenoids and/or β-carotene and β-cryptoanthin content in peach genotypes belonging to golden-yellow, orange-yellow, and dull-orange groups. The authors highlighted that the β-cryptoanthin was strongly correlated with flesh-color value a* and moderately correlated with the b* value, while a low correlation was found between β-carotene and color parameters [54]. Our results, on the contrary, showed a low correlation or non-correlation between total carotenoids and colorimetric variables in the analyzed cultivars (data not shown).

Phenolic compounds are secondary plant metabolites that act as antioxidant and pro-oxidant agents [3,55]. Many studies have focused on determining the phenolic content of peach extracts; results showed that the content of total polyphenols in peaches is higher in the skin than in the flesh and is correlated with the cultivar and the different stages of ripening [3,24,52,56,57,58].

The content of total polyphenols, determined by Folin–Ciocalteu’s method, of 32 peach cultivars (yellow and white fruits) and 52 nectarine cultivars (yellow and white fruits) is reported in Table 6. The average content of total polyphenols of yellow-flesh nectarines was 0.640 mg of GAE/g^−1^ FW, with significant differences depending on the cultivars. ‘Gianna Laura Dolce’, ‘Max’, ‘Spring Red’, and ‘Stark Redgold’ cultivars had the highest values, whereas ‘Orion’ (0.248 mg of GAE/g^−1^) has a much lower content than any other cultivar. The mean content of total polyphenols lies between 0.929 and 0.192 mg g^−1^ of FW for white-flesh nectarines in the following increasing order: ‘Maria Linda’ > ‘Silver Giant’ > ‘Silver Star’ > ‘Caldesi 2010’ > ‘Caldesi 2000’ > ‘Maria Anna’ > ‘Silver Ray’ > ‘Caldesi 2020’ (Table 6). A large difference in polyphenol contents has also been reported in peach cultivars. 

In the case of yellow-flesh peaches, the concentration of polyphenols ranged from 1.951 to 0.250 mg of GAE g^−1^ FW. The highest value was observed in ‘Flavorcrest’, followed by ‘Vistarich’—1.164 and 1.951 mg of GAE g^−1^ FW, respectively—and decreased to 0.272 and 0.250 in ‘Maria Silvia’ and ‘Padana’, respectively. The average value of TPC of white-flesh fruits was 0.520 mg of GAE g^−1^ FW, and the greatest TPC was found in ‘Rosa del West’, whereas the lowest values were found in ‘Tardivo Zuliani’ (Table 6). 

TPC values recorded in our study were in agreement with the representative values reported in the literature [3,24,52]. Gil et al. [24] reported an average value of 45.9 mg/100 g FW (range from 102.3 to 13.6 mg/100) in white-flesh nectarines and 32.0 mg/100 g (values between 54 and 17.5 mg/100 g Fw) in yellow-flesh nectarines; in yellow-flesh and white-flesh peaches, the authors recorded TPC values of 354 mg/kg and 534 mg/kg, respectively. Vizzotto and coworkers [52], by analyzing 19 peach cultivars, observed total polyphenol contents of 252 and 190 mg/100 g FW in white-flesh and yellow-flesh fruits, respectively. In the study of Mitic et al. [58], the TPC ranged from from 0.55 to 4.01 mg GAE g^−1^ FW for peach (average content 1.88 mg/g) and from 0.93 to 1.83 mg GAE g^−1^ FW for nectarine (average content 1.28 mg/g). Recently, Mihaylova et al. [57] measured total polyphenols in peach and nectarine pulp fruits in the range 104.9–34.1 mg GAE/100 g FW.

Two-way ANOVA analysis, performed on the mean TPC values, showed no differences on two factors: type (peaches vs. nectarines) and flesh color (yellow vs. white). In fact, the results were very close to each other (Table S2f). However, analysis of TPC values in the individual groups (yellow peaches, white peaches, yellow nectarines, and white nectarines) showed marked intervarietal variability. Our results suggest the important role played by genotype in determining polyphenol content, as reported by other authors [3,49,52,59,60].

On the other hand, work by Gil et al. [20] and Cantin et al. [45] reported a higher value of total polyphenols in white nectarines than in yellow-flesh peaches and in nectarines than in peaches. Di Vaio et al. [59], instead, found lower TPC values in nectarines, either yellow and white, compared with peaches.

The antioxidant activities obtained by the DPPH method for the fruit extracts are presented in Table 6. Our results showed that white-flesh nectarines presented an average antioxidant value higher than that in yellow-flesh nectarines—33.72 mg/g and38.4 mg/g, respectively. In yellow-flesh nectarines, ‘Maeba Top’ was the one with the lowest activity, followed by ‘Gioia’ and ‘Honey Kist’. In white-flesh nectarines, DPPH values ranged from 55.12 mg mL^−1^ (‘Caldesi 2000’) to 19.39 mg mL^−1^ (‘Maria Anna’). As in the case of nectarines, white-flesh peaches showed an average antioxidant value higher than that in yellow-flesh peaches: 39.7 mg/mL vs. 44.2 mg/mL, respectively. Antioxidant capacity of yellow-flesh peaches was in the range 120.3–13.47 mg mL^−1^; the highest values have been registered in ‘Symphonie’ and ‘Zee Lady’ cvs. In white-flesh peaches, the total antioxidant activity ranked as follows: ‘Michelini’ > ‘Maria Regina’ > ‘Tardivo Zuliani’ > ‘Maria Bianca’ (Table 6).

Two-way ANOVA analysis performed on the mean antioxidant activity showed no differences on two two-level factors: type (peaches vs. nectarines) and flesh color (yellow vs. white). In fact, the results were very close to each other (Table S2g). However, analysis in the individual group (yellow peaches, white peaches, yellow nectarines, and white nectarines) showed marked intervarietal variability. No clear trend in phenol content and antioxidant capacity was found among both white- and yellow-fleshed nectarines and peaches. It is the individual cultivar that plays an important role. For example, “Alitop” (yellow-fleshed nectarines) and “Caldesi 2020” (white-fleshed nectarines) and ‘Michelini’ (white-fleshed peaches) and ‘Red Coast’ (yellow-fleshed peaches) had similar phenolic content but different antioxidant activity.

Our results do not agree with previous work, where a marked relationship was observed between TPC and antioxidant activity [3,28,55]. As reported in the literature, different reasons can explain the unclear relation between total phenols and antioxidant activity [61]: (i) the total phenols did not include all antioxidants, (ii) various interactions between antioxidant active compounds may occur, and (iii) in addition, synergistic effect between antioxidant vitamins and phenolic compounds can affect the antioxidant activity.

### 2.4. HPLC-DAD-MS Analysis

HPLC-DAD-MS analysis evidenced the presence of four main compounds in all examined cultivars: hydroxycinnamic acid derivatives (neochlorogenic acid and chlorogenic acid) and flavan-3-ols (catechin and epicatechin). Detection of chlorogenic acid, neochlorogenic acid, catechin, and epicatechin was in accordance with the literature [3,28,51]. Phenolic concentration of the above-mentioned phenols is reported in Tables S5 and S6. Other derivates were detected at very low concentration and were not identified (not reported).

The total content of phenolics in yellow-flesh nectarine ranged from 15.5 to 379.5 mg/kg in ‘Silvana’ and ‘Orion’, respectively, whereas in white-flesh nectarines it ranged from 59.6 to 494.3 mg/kg in ‘Kaweah’ and ‘Silver Star’, respectively. Cultivars of yellow-flesh nectarines and white-flesh nectarines exhibited an average content of chlorogenic acid higher than neochlorogenic acid, as reported in the literature [28]. Among the examined cultivars, the yellow-flesh nectarine ‘Morsiani 51’ exhibited the highest content in chlorogenic acid, while the cultivar ‘Max’ had the highest content of neochlorogenic acid, 128.27 mg/kg. White-flesh nectarine cultivar ‘Silver Star’ showed the highest content in chlorogenic and neochlorogenic acid: 184.67 and 108.08 mg/kg, respectively (Table S5).

In regard to the flavan-3-ol content of the nectarine cultivars, epicatechin was detected in all yellow-flesh and white-flesh nectarines with an average content higher for yellow-flesh nectarines: 23.46 mg/kg and 19.40 mg/kg, respectively. On the contrary, catechin was not detected in all analyzed cultivars, but its average content was 58.69 mg/kg in yellow-flesh nectarines and 63.21 mg/kg in white-flesh nectarines. Among the yellow-flesh nectarines, ‘Lady Star’ was the richest in this compound, with 132.57 mg/kg, while the white-flesh cultivar ‘Maria Anna’ had a content of 113.83 mg/kg (Table S5). 

The total content of phenolics in yellow-flesh peaches ranged from 23.4 to 425.45 mg/kg in ‘Glohaven’ and ‘Cotogna del Poggio’ (traditional varieties), respectively, whereas in white-flesh peaches it ranged from 35.6 to 313.5 mg/kg in ‘Maria Bianca’ and ‘Rosa del West’, respectively (Table S6). In most of the peach cultivars, the average content of flavan-3-ols was higher than the average content of hydroxycinnamates. The average content of chlorogenic acid was higher than the average content of neochlorogenic, either in yellow-flesh or in white-flesh peaches. Among yellow-flesh peaches, ‘Summer Rich’ presented the highest level of chlorogenic acid, with 116.22 mg/kg, while ‘Maria Marta’ was the richest in neochlorogenic acid, with 50.58 mg/kg. Among white-flesh peaches, ‘Greta’ exhibited the highest content in chlorogenic acid, 63.64 mg/kg, and in neochlorogenic acid, 46.67 mg/kg (Table S6). Epicatechin was detected in all yellow-flesh and white-flesh peaches, with an average content in yellow-flesh peaches equal to white-flesh peaches, 25.34 mg/kg and 22.75 mg/kg, respectively, with white-flesh cv ‘Weinberger’ exhibiting the highest content, 37.29 mg/kg. As in nectarines, catechin was not present in all yellow-flesh and white-flesh peaches; among yellow-flesh peaches, ‘Zee Lady’ exhibited the highest content, 132.71 mg/kg, while ‘Rosa del West’ was the richest in catechin among white-flesh peaches, with 117.24 mg/kg (Table S6). 

**Figure 5:**
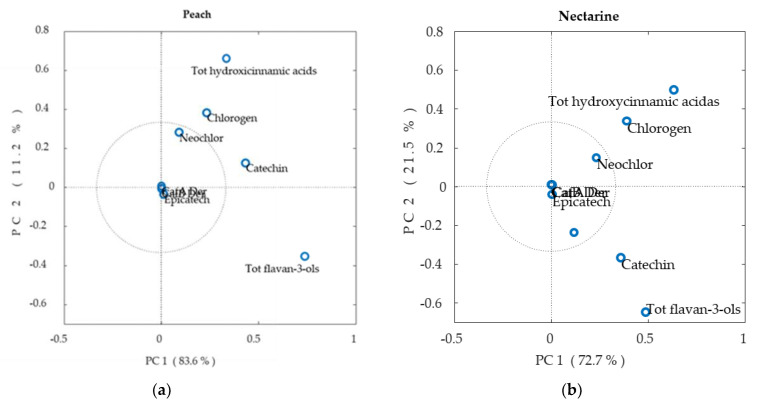
Principal component analysis (PCA) diagrams of HPLC data of peach (**a**) and nectarine (**b**) samples.

The values of peach and nectarine phenolic content were analyzed separately by PCA analysis. The results revealed a maximum of three principal components (PCs), and PC1 and PC2 explained over 90% of the variance in the datasets for both peaches and nectarines. PC1 exceeded 83% of variance in peaches and 70% in nectarines, whereas PC2 explained 21% and 11% of the variance in nectarines and peaches, respectively (Figure 5). In peaches, PC1 is dominated by catechins, while hydroxycinnamic acids have stronger loads on PC2. In nectarines instead, catechin and neochlorogenic acid and chlorogenic acid have similar loads along either PC1 or PC2. 

In general, the neochlorogenic acid and chlorogenic acid content described by the PCA analysis showed differences only between peaches and nectarines, while no significant differences were observed between yellow and white cultivars because the variances of the data were similar. The PCA analysis did not provide additional information compared with the two-way ANOVA, which showed similar results (Table S2h–m).

## 3. Materials and Methods

### 3.1. Chemicals, Standards, and Reagents

Neochlorogenic and chlorogenic acid, epicatechin, and catechin standards, Folin–Ciocalteu reagent, 2,2-diphenyl-1-picrylhydrazyl (DPPH), and other chemicals used were of analytical grade and were purchased from the Sigma Chemical Co. (St. Louis, MO, USA). The water used in sample preparation, solutions, and analysis was obtained with a Milli-Q water purification system by Millipore (Bedford, MA, USA).

### 3.2. Plant Materials

The study was conducted on fruits of 32 peach cultivars (yellow and white fruits) and 52 nectarine cultivars (yellow and white fruits) (Table S1). The fruits were obtained from several commercial companies located in Cesena (Italy) between June and September (depending on the ripening period). Once harvested, the fruits were transported to the laboratory and then processed. For each cultivar, three lots of six fruits of uniform section and free from defects were sampled and processed in three replications. All fruits were peeled, had pits removed, had flesh chopped into small pieces, and were processed by an electric blender, obtaining homogeneous peach-puree samples. The samples were divided into sub-samples and kept at −20 °C until analyzed.

### 3.3. Quality Parameters

Five grams of peach puree were centrifuged at 8000 rpm for 15 min at +4 °C with a centrifuge Varian T21, and the juice was analyzed for the following quality indices: soluble solid content (SSC), titratable acidity (TA), pH, and ripening index (RI). SSC were measured with a digital refractometer (Hanna HI96811; HANNA Instruments, Padova, Italy), and expressed as °Brix; pH values were determined using a digital pH meter (Crison Basic 20) calibrated with pH 4 and 7 buffers; TA values (g malic acid L^−1^) were determined according to Capocasa et al. [62]: 10 mL of juice was diluted with distilled water and titrated to pH 8.2 against 0.1 N NaOH. On values of SSC and TA were calculated ripening index values (RI) as an SSC/TA ratio.

Peach puree color was measured using a Minolta CR-200 chromatometer (Minolta, Ramsey, NJ, USA), determining L* (brightness; 0 = black, 100 = white), a* (red = positive values, green = negative values), and b* (yellow = positive values, blue negative values) values [63,64]. Next, the values of a* and b* were used to calculate the color index as an a/b ratio. Three measurements of quality indices were made for all cultivars.

### 3.4. Determination of Total Carotenoids, Total Phenolic, and DPPH Assay

The peach samples (5 g) were homogenized with 10 mL of extract solution. The extract solution was composed of ethanol/acidified water (pH 2.0, by formic acid) (7/3, *v*/*v*) [65]. Samples were centrifuged at 3500 rpm and 4 °C for 15 min. Two subsequent extraction steps were undertaken and both supernatants were combined and used for the determination of total phenolic content and antioxidant activity. 

Total polyphenol contents were determined using a Folin–Ciocalteu assay as reported by [66], using gallic acid as standard. A diluted 50 mL of the extracted samples (5 mg/mL) were placed in a 25 mL flask and diluted with methanol: water 80:20% *v*/*v* to 15 mL. Then, 2.5 mL of Folin–Ciocalteu reagent (10% *v*/*v* water solution) was added and the mixture was shaken for 30 s. Afterward, 5 mL of a saturated NaCO3–water solution was added and the mixture was left at room temperature for 1 h. The absorbance of the colored reaction product was read at 730 nm using a Varian UV-visible spectrophotometer Cary 50 Scan and using distilled water as blank. Results were expressed as mg of gallic acid equivalent per g of fresh weight (mg GAE/g FW) using pure gallic acid as standard (Sigma-Aldrich, Milan, Italy). The total phenolic compounds quantification was performed in triplicate. 

For carotenoids extraction, peach samples (5 g) were mixed with 5 mL of 90% acetone; then, the extracts were centrifugate at 4000× *g* for 5 min. The supernatants were collected and placed into a new tube. Total carotenoid amount was determined spectrophotometrically in 90% acetone extracts, as previously described [66]. The results were expressed as mg per g FW of sample. The analysis was performed in triplicate. 

The free-radical scavenging activity was determined using the 1,1 diphenyl-2-picryl-hydrazyl (DPPH) reagent for samples, as according to Petruccelli et al. [64]. Then, 0.1 mL of appropriately diluted methanolic extract (2.5–0.16 mg/mL) was added to 1 mL of freshly prepared methanolic DPPH solution (0.9 × 10^−4^ M) and stirred. The decolorizing process was recorded at the beginning (control) and after 20 min (sample) of reaction at 517 nm. Antiradicalic activity was calculated according to the following formula:

DPPH inhibition% = (control absorbance − sample absorbance)/control absorbance) × 100.

Values were expressed as mg/mL of extracts necessary to inhibit 50% of DPPH free radicals (I50). Measurements were carried out in triplicate.

### 3.5. Polyphenols HPLC-MS Analysis

The analyses were carried out using an HP-1100 liquid chromatograph equipped with a DAD detector (Agilent-Technologies, Palo Alto, CA, USA). The column was a Zorbax SB-Aq, 3 × 150 mm, 3.5 µm from Agilent Technologies. The mobile phase was (A) water pH 3.2 acidified by formic acid and (B) acetonitrile. The following multi-step linear gradient was applied: from 95% of A to 85% in 10 min, which was maintained for 5 min, from 85% A to 75% A in 8 min, which was maintained for 5 min, to 0% A in 5 min, which was maintained for 5 min. Total time of analysis was 38 min, flow rate was 0.4 mL/min, and oven temperature was 30 ± 0.5 °C. UV/Vis spectra were recorded in the 190–600 nm range, and the chromatograms were acquired at 280, 284, 330, and 350 nm. The above HPLC system was interfaced with an MSD API Electrospray (Agilent Technologies, Palo Alto, CA, USA) operating in negative and positive ionization mode. Gas temperature was 350 °C, flow rate 10.0 L/min, nebulizer pressure 30 psi, quadrupole temperature 300 °C, capillary voltage 3500 V, and fragmentor 120 eV. Identification of phenolic compounds was carried out by comparing their UV-vis spectra (280–350 nm) and mass spectra with the available literature and retention times relative to available external standards. Quantification of individual polyphenolic compounds was directly performed by HPLC-DAD using a five-point regression curve (r^2^ ≥ 0.998) on the basis of authentic standards. Calibration was performed at the wavelength of the maximum UV-vis absorbance. In particular, flavan-3-ols were calibrated at 284 nm, and hydroxycinnamic derivatives were calibrated at 330 nm. The determinations of the polyphenol contents were carried out in triplicate; the results are given as means, and the standard error was <5%.

### 3.6. Sugar Content Analysis

Flesh samples were analyzed for content of soluble carbohydrate (sucrose, glucose, fructose, sorbitol) according to (64). The extraction required the addition of 12 mL of bidistilled water (pH 7) to 20 mg of freeze-dried fruit puree, The solution was stirred for 1 h, then centrifugerd for 5 min at 10,000× *g* at 4°C. The supernatant was filtered through a 0.20-µm filter (Millex-FG; Merck Millipore Co., Darmstadt, Germany). Analysis was perforned by HPLC equipped with a Shodex Sugar Series SC-1011 8 mm × 300 mm column (Showa Denko Europe Gmbh, Gerrmany), and a precolumn Showa Denko Europe Gmbh, Gerrmany, with a Guard Pack Insert Sugar Pack II (Waters, Milford, MA, USA) and installed in the LC system Flexar (Perkin Elmer). The mobile phase was water Milli-Q grade, at a flow rate of 0.5 mL/min^−1^. Individual carbohydrates were identified and quantified by comparison of retention times with those of authentic carbohydrate standards (Sigma-Aldrich, St. Louis, MO, USA). Analyses were performed in triplicate.

### 3.7. Statistical Analysis

One-way analysis of variance (ANOVA) was used for testing the equality of means of single variables against the effect of kind (peach vs. nectarine) or color (yellow vs. white) separately. Two-way ANOVA was used for testing the equality of means of single variables, considering the effects of kind and color simultaneously. The effect of their interaction was also included in the model. All significant differences at the 5% level or better were signaled. PCA analysis was carried out on the HPLC data of peach and nectarine cultivars. All analysis was carried out by means of open-source software JASP^®^ (The JASP team, University of Amsterdam, Amsterdam, The Netherlands).

## 4. Conclusions

In this work, fruit quality parameters and chemical composition were determined in 84 cultivars of *Prunus persica* L. Considerable quantitative intervarietal variability was observed in peach and nectarine cultivars and different flesh types (yellow and white-fleshed cvs). In particular, the nectarine cultivars showed higher diversity for SSC and titratable acidity and sugar values than peaches. The mean concentration of carotenoid, found in yellow-flesh types (peaches and nectarines), was almost double that observed in the white-fleshed fruits. Yellow-fleshed nectarines had a higher average antioxidant value than white-fleshed fruits and were on average richer in neo-chlorogenic acid than peaches. Results obtained in this study, as reported by previous studies [52,57], confirm the important role played by genotype in determining the quality attribute parameters, sugar levels, and availability of bioactive compounds. In addition, our results on the content of primary and secondary metabolites corroborate the knowledge on the nutritional value of peach fruits, which act as important protective factors of human health.

## Figures and Tables

**Table 1:** Values of Quality Indices SSC (%, total soluble solids content), TA (g malic acid L^−1^, titratable acidity), pH, RI (ripening index), and flesh color (L*, a*, b*) of yellow and white peaches.

Yellow-Flesh cvs	SSC	TA	pH	RI Index	L*	a*	b*	Color Index
Elegant Lady	11.78 ± 0.19	7.11 ± 0.02	3.81 ± 0.02	1.65 ± 0.03	53.06 ± 0.05	0.37 ± 0.05	25.15 ± 0.02	0.01 ± 0.00
Fayette	12.25 ± 0.18	8.44 ± 0.02	3.86 ± 0.01	1.45 ± 0.02	55.91 ± 0.06	10.15 ± 0.05	11.75 ± 0.01	0.86 ± 0.00
Flavorcrest	11.56 ± 0.21	9.72 ± 0.01	3.92 ± 0.02	1.19 ± 0.02	50.45 ± 0.06	−1.31 ± 0.01	20.85 ± 0.01	−0.06 ± 0.00
Glohaven	12.83 ± 0.15	10.39 ± 0.01	4.05 ± 0.01	1.23 ± 0.01	44.36 ± 0.13	2.55 ± 0.08	26.33 ± 1.73	0.09 ± 0.00
Grenat	12.43 ± 0.26	9.19 ± 0.01	4.62 ± 0.01	1.35 ± 0.02	52.12 ± 0.04	−0.90 ± 0.01	21.02 ± 0.02	−0.04 ± 0.00
Guglielmina	12.16 ± 0.56	9.98 ± 0.06	4.28 ± 0.02	1.22 ± 0.05	45.31 ± 0.85	2.72 ± 0.15	25.25 ± 0.52	0.11 ± 0.00
Kaweah	13.27 ± 0.25	8.16 ± 0.05	3.90 ± 0.01	1.62 ± 0.01	51.01 ± 0.61	−1.12 ± 0.11	19.98 ± 0.56	−0.06 ± 0.01
Lara Star	12.33 ± 0.30	10.94 ± 0.05	3.97 ± 0.01	1.13 ± 0.03	45.14 ± 0.02	6.70 ± 0.10	12.21 ± 0.17	0.55 ± 0.00
Lizbeth	11.76 ± 0.21	7.94 ± 0.01	3.62 ± 0.02	1.48 ± 0.02	52.51 ± 0.15	−0.60 ± 0.08	17.81 ± 0.08	−0.03 ± 0.00
Maria Marta	12.55 ± 0.40	8.31 ± 0.02	4.42 ± 0.01	1.50 ± 0.04	52.30 ± 0.02	0.43 ± 0.05	27.67 ± 0.54	0.01 ± 0.00
Maria Silvia	12.60 ± 0.20	8.05 ± 0.03	3.56 ± 0.02	1.56 ± 0.03	56.97 ± 0.05	−1.18 ± 0.05	19.38 ± 0.01	−0.06 ± 0.00
Padana	10.25 ± 0.22	8.99 ± 0.02	4.00 ± 0.01	1.13 ± 0.03	45.50 ± 0.16	2.96 ± 0.04	23.78 ± 1.39	0.12 ± 0.01
Red Coast	11.30 ± 0.26	10.26 ± 0.04	3.99 ± 0.02	1.11 ± 0.02	45.95 ± 0.02	4.48 ± 0.04	32.64 ± 0.15	0.14 ± 0.00
Redhaven	11.28 ± 0.24	8.86 ± 0.02	4.43 ± 0.02	1.27 ± 0.02	49.88 ± 0.02	1.14 ± 0.05	22.56 ± 0.07	0.05 ± 0.00
Red Valley	12.36 ± 0.32	7.54 ± 0.01	3.52 ± 0.03	1.64 ± 0.05	49.86 ± 0.02	1.14 ± 0.04	22.55 ± 0.08	0.05 ± 0.00
Rich Lady	13.36 ± 0.21	12.09 ± 0.07	3.76 ± 0.01	1.10 ± 0.07	49.34 ± 0.14	3.78 ± 0.02	12.82 ± 0.09	0.29 ± 0.01
Rome Star	13.86 ± 0.15	7.12 ± 0.02	4.21 ± 0.02	1.95 ± 0.03	47.73 ± 0.04	1.68 ± 0.09	25.67 ± 0.37	0.06 ± 0.00
Summer Rich	13.33 ± 0.25	11.05 ± 0.02	4.14 ± 0.03	1.21 ± 0.03	39.67 ± 0.05	5.69 ± 0.14	28.74 ± 0.12	0.19 ± 0.00
Suncrest	11.23 ± 0.32	8.36 ± 0.03	3.59 ± 0.01	1.34 ± 0.03	48.51 ± 0.04	2.51 ± 0.06	28.01 ± 0.01	0.09 ± 0.00
Symphonie	11.53 ± 0.30	9.00 ± 0.01	3.37 ± 0.02	1.28 ± 0.03	101.22 ± 0.03	−1.41 ± 0.10	16.05 ± 0.83	0.09 ± 0.04
Vistarich	9.38 ± 0.34	7.99 ± 0.03	3.54 ± 0.03	1.17 ± 0.05	55.68 ± 0.55	10.05 ± 0.25	11.76 ± 0.09	0.85 ± 0.01
Zee Lady	12.95 ± 0.15	10.95 ± 0.03	3.69 ± 0.01	1.18 ± 0.02	44.76 ± 0.03	1.65 ± 0.08	23.73 ± 0.15	0.07 ± 0.00
**Percoca peaches**	**SSC**	**TA**	**pH**	**RI index**	L*	a*	b*	**Color index**
Babygold 7	9.90 ± 0.10	5.25 ± 0.14	3.96 ± 0.02	1.88 ± 0.03	48.45 ± 0.06	0.39 ± 0.02	22.28 ± 0.15	0.02 ± 0.00
Babygold 9	11.14 ± 0.18	6.49 ± 0.02	4.03 ± 0.07	1.72 ± 0.03	43.62 ± 0.17	3.39 ± 0.06	24.09 ± 1.40	0.14 ± 0.00
Carson	12.92 ± 0.16	7.59 ± 0.02	4.21 ± 0.02	1.70 ± 0.02	45.17 ± 0.02	5.43 ± 0.13	32.75 ± 0.20	0.17 ± 0.00
Cotogna del Poggio	13.27 ± 0.14	5.81 ± 0.05	4.26 ± 0.01	2.28 ± 0.04	50.43 ± 0.18	1.67 ± 0.10	24.13 ± 0.24	0.07 ± 0.00
**White-flesh cvs**	**SSC**	**TA**	**pH**	**RI index**	L*	a*	b*	**Color index**
Greta	13.23 ± 0.25	8.12 ± 0.03	3.83 ± 0.02	1.63 ± 0.02	46.23 ± 0.01	3.12 ± 0.05	29.93 ± 0.07	0.10 ± 0.00
Maria Bianca	11.47 ± 0.30	8.10 ± 0.02	3.73 ± 0.02	1.41 ± 0.02	49.69 ± 0.01	1.87 ± 0.07	21.19 ± 0.04	0.09 ± 0.00
Maria Regina	11.50 ± 0.30	9.30 ± 0.01	3.77 ± 0.01	1.24 ± 0.02	40.02 ± 0.09	5.44 ± 0.07	26.26 ± 0.33	0.20 ± 0.00
Michelini	12.36 ± 0.36	6.63 ± 0.02	3.53 ± 0.02	1.85 ± 0.03	51.37 ± 0.05	1.44 ± 0.08	25.19 ± 0.06	0.06 ± 0.00
Rosa del West	9.53 ± 0.30	7.38 ± 0.01	3.72 ± 0.02	1.29 ± 0.02	49.35 ± 0.01	3.79 ± 0.01	12.82 ± 0.02	0.29 ± 0.00
Tardivo Zuliani	11.9 ± 0.10	9.76 ± 0.01	3.69 ± 0.01	1.22 ± 0.04	42.60 ± 0.12	2.96 ± 0.09	20.03 ± 0.68	0.15 ± 0.00

The data are presented as the mean (*n* = 3) ± S.D.

**Table 2:** Values of Quality Indices SSC (°Brix, total soluble solids content), TA (g malic acid L^−1^, titratable acidity), pH, RI (ripening index), and flesh color (L, a*, b*) of yellow and white nectarines.

Yellow-Flesh cvs	SSC	TA	pH	RI Index	L*	a*	b*	Color Index
Alitop	12.23 ± 0.32	7.33 ± 0.02	4.32 ± 0.02	1.67 ± 0.04	51,25 ± 0.01	−1.42 ± 0.10	18.47 ± 0.16	−0.08 ± 0.00
Alma	10.93 ± 0.32	11.19 ± 0.04	3.99 ± 0.02	0.98 ± 0.02	41.75 ± 0.05	3.36 ± 0.17	25.85 ± 0.17	0.13 ± 0.00
Amiga	11.46 ± 0.35	7.16 ± 0.11	3.49 ± 0.10	1.60 ± 0.08	53.74 ± 0.08	−2.39 ± 0.19	9.59 ± 0.20	−0.25 ± 0.01
Antares	12.03 ± 0.40	7.98 ± 0.05	3.63 ± 0.02	1.51 ± 0.07	43.05 ± 0.17	5.98 ± 0.09	24.88 ± 0.22	0.24 ± 0.00
August Red	12.27 ± 0.46	10.01 ± 0.03	3.61 ± 0.01	1.22 ± 0.05	45.43 ± 0.18	2.99 ± 0.13	25.99 ± 0.14	0.11 ± 0.00
Big Top	13.95 ± 0.14	7.82 ± 0.07	4.47 ± 0.02	1.78 ± 0.01	57.29 ± 0.61	−1.16 ± 0.18	15.18 ± 0.16	−0.05 ± 0.01
Claudia	11.36 ± 0.29	6.10 ± 0.03	3.51 ± 0.02	1.86 ± 0.06	48.28 ± 0.44	2.75 ± 0.09	31.48 ± 0.24	0.09 ± 0.00
Diamond Princess	12.33 ± 0.41	9.89 ± 0.12	3.73 ± 0.08	1.25 ± 0.07	57.01 ± 0.06	−1.16 ± 0.18	19.25 ± 0.12	−0.06 ± 0.01
Diamond Ray	13.33 ± 0.42	11.24 ± 0.06	3.82 ± 0.02	1.18 ± 0.03	52.10 ± 0.94	−0.72 ± 0.20	20.22 ± 0.13	−0.03 ± 0.01
Fire Top	11.03 ± 0.16	8.64 ± 0.02	3.88 ± 0.01	1.28 ± 0.01	55.94 ± 0.03	0.24 ± 0.10	24.79 ± 0.15	0.09 ± 0.00
Gianna Laura Dolce	13.33 ± 0.29	8.60 ± 0.01	4.73 ± 0.02	1.55 ± 0.03	57.09 ± 0.16	10.24 ± 0.12	11.86 ± 0.22	0.86 ± 0.00
Gioia	9.63 ± 0.25	6.08 ± 0.01	3.94 ± 0.03	1.58 ± 0.04	48.21 ± 0.02	1.07 ± 0.11	24.25 ± 0.20	0.04 ± 0.00
Guerriera	11.81 ± 0.11	12.14 ± 0.07	3.55 ± 0.02	0.97 ± 0.04	53.69 ± 0.01	−0.76 ± 0.09	19.46 ± 0.15	−0.04 ± 0.00
Honey Kist	14.38 ± 0.19	7.74 ± 0.01	3.50 ± 0.01	1.86 ± 0.03	51.90 ± 0.10	−0.36 ± 0.11	17.53 ± 0.19	−0.02 ± 0.00
Honey Royale	15.34 ± 0.12	9.97 ± 0.02	4.21 ± 0.01	1.54 ± 0.01	55.47 ± 0.05	−0.67 ± 0.09	17.31 ± 0.24	−0.04 ± 0.00
Independence	9.47 ± 0.41	12.29 ± 0.12	3.87 ± 0.01	0.77 ± 0.05	59.21 ± 0.23	−0.55 ± 0.09	26.49 ± 0.18	−0.02 ± 0.00
Lady Erika	13.20 ± 0.20	5.35 ± 0.02	4.15 ± 0.02	2.46 ± 0.03	50.30 ± 0.11	0.61 ± 0.14	25.70 ± 0.25	0.02 ± 0.00
Lady Star	12.03 ± 0.24	8.30 ± 0.02	3,76 ± 0.03	1.45 ± 0.03	64.99 ± 0.05	7.82 ± 0.06	25.56 ± 0.12	0.30 ± 0.00
Licinia	9.01 ± 0.20	5.94 ± 0.03	3.77 ± 0.02	1.51 ± 0.04	56.49 ± 0.01	−1.47 ± 0.14	16.77 ± 0.15	−0.09 ± 0.00
Maeba Top	11.66 ± 0.22	10.68 ± 0.01	3.70 ± 0.01	1.09 ± 0.02	49.92 ± 0.06	−1.20 ± 0.06	16.69 ± 0.24	−0.07 ± 0.00
Maria Aurelia	9.27 ± 0.11	13.99 ± 0.03	3.60 ± 0.02	0.66 ± 0.01	48.33 ± 0.12	2.26 ± 0.28	17.50 ± 0.18	0.13 ± 0.01
Maria Camilla	11.59 ± 0.21	9.53 ± 0.01	4.02 ± 0.02	1.22 ± 0.02	46.10 ± 0.06	4.01 ± 0.17	30.27 ± 0.13	0.13 ± 0.00
Maria Carla	11.80 ± 0.20	7.36 ± 0.07	3.72 ± 0.02	1.60 ± 0.03	51.12 ± 0.03	1.39 ± 0.20	26.23 ± 0.10	0.05 ± 0.00
Maria Dolce	14.53 ± 0.42	10.24 ± 0.08	4.26 ± 0.01	1.42 ± 0.02	60.06 ± 0.13	8.97 ± 0.11	21.33 ± 0.24	0.042 ± 0.00
Maria Dorata	14.05 ± 0.15	7.77 ± 0.03	4.01 ± 0.01	1.81 ± 0.02	51.78 ± 0.26	−1.04 ± 0.08	15.40 ± 0.15	−0.07 ± 0.00
Maria Laura	12.43 ± 0.30	8.03 ± 0.01	3.55 ± 0.02	1.55 ± 0.04	55.39 ± 0.10	−0.62 ± 0.09	17.32 ± 0.15	−0.03 ± 0.00
Max	13.01 ± 0.20	6.36 ± 0.01	3.70 ± 0.01	2.04 ± 0.03	46.65 ± 0.04	3.42 ± 0.16	33.83 ± 0.16	0.10 ± 0.00
Morsiani 60	13.54 ± 0.24	9.95 ± 0.02	3.75 ± 0.01	1.36 ± 0.02	51.07 ± 0.40	0.18 ± 0.07	24.84 ± 0.11	0.007 ± 0.00
Morsiani 51	14.01 ± 0.21	8.44 ± 0.02	3.99 ± 0.01	1.66 ± 0.03	47.60 ± 0.12	1.64 ± 0.08	23.12 ± 0.08	0.07 ± 0.00
Nectaross	11.18 ± 0.22	9.23 ± 0.02	3.05 ± 0.02	1.21 ± 0.02	55.24 ± 0.05	0.57 ± 0.13	27.78 ± 0.22	0.02 ± 0.00
Orion	15.05 ± 0.13	13.31 ± 0.03	3.98 ± 0.03	1.13 ± 0.01	49.08 ± 0.05	2.87 ± 0.15	30.66 ± 0.13	0.09 ± 0.00
Red Jewel	12.82 ± 0.17	13.37 ± 0.02	3.83 ± 0.02	0.96 ± 0.01	53.57 ± 0.33	−0.26 ± 0.07	9.68 ± 0.18	−0.03 ± 0.00
Silvana	12.31 ± 0.16	9.64 ± 0.02	3.53 ± 0.01	1.28 ± 0.02	49.64 ± 0.03	0.31 ± 0.10	18.20 ± 0.15	0.02 ± 0.00
Spring Bright	11.57 ± 0.17	11.26 ± 0.04	3.57 ± 0.01	1.03 ± 0.01	97.38 ± 0.03	−0.60 ± 0.14	23.27 ± 0.09	−0.02 ± 0.04
Spring Red	11.50 ± 0.36	9.56 ± 0.03	3.58 ± 0.02	1.20 ± 0.04	55.36 ± 0.04	−0.76 ± 0.10	20.29 ± 0.09	−0.04 ± 0.00
Star Bright	13.50 ± 0.30	8.03 ± 0.02	3.34 ± 0.02	1.68 ± 0.04	53.88 ± 0.05	−0.28 ± 0.07	22.52 ± 0.20	−0.01 ± 0.00
Stark Redgold	14.07 ± 0.20	13.78 ± 0.11	3.64 ± 0.03	1.02 ± 0.04	43.32 ± 0.32	4.53 ± 0.20	30.13 ± 0.16	0.15 ± 0.00
Summer Grand	11.56 ± 0.25	14.01 ± 0.03	3.60 ± 0.01	0.82 ± 0.02	52.35 ± 0.02	−1.15 ± 0.07	16.35 ± 0.12	−0.07 ± 0.00
Superior Super Star	11.40 ± 0.19	5.53 ± 0.02	3.91 ± 0.02	2.06 ± 0.04	52.73 ± 0.01	0.31 ± 0.08	24.08 ± 0.13	0.01 ± 0.00
Sweet Lady	16.33 ± 0.30	15.01 ± 0.01	3.34 ± 0.01	1.09 ± 0.02	45.45 ± 0.04	3.81 ± 0.10	27.39 ± 0.22	0.14 ± 0.00
Sweet Red	14.04 ± 0.15	13.08 ± 0.06	3.90 ± 0.03	1.07 ± 0.05	49.67 ± 0.04	0.44 ± 0.11	23.33 ± 0.21	0.02 ± 0.00
Vega	10.99 ± 0.19	6.61 ± 0.02	3.61 ± 0.01	1.66 ± 0.03	53.02 ± 0.18	0.38 ± 0.12	25.15 ± 0.17	0.01 ± 0.00
Venus	10.80 ± 0.20	10.55 ± 0.06	3.62 ± 0.03	1.02 ± 0.02	45.15 ± 0.15	3.22 ± 0.20	27.37 ± 0.15	0.12 ± 0.00
Weinberger	10.88 ± 0.34	6.74 ± 0.03	3.99 ± 0.01	1.61 ± 0.04	41.46 ± 0.07	2.18 ± 0.14	24.22 ± 0.17	0.09 ± 0.00
**White-flesh cvs**	**SSC**	**TA**	**pH**	**RI index**	L*	a*	b*	**Color index**
Caldesi 2000	11.55 ± 0.21	7.48 ± 0.03	3.94 ± 0.01	1.49 ± 0.03	89.29 ± 0.07	0.54 ± 0.10	5.72 ± 0.15	0.09 ± 0.01
Caldesi 2010	10.49 ± 0.14	8.33 ± 0.02	3.53 ± 0.03	1.26 ± 0.02	81.09 ± 0.12	1.59 ± 0.08	33.05 ± 0.17	0.05 ± 0.00
Caldesi 2020	12.34 ± 0.25	9.69 ± 0.01	3.74 ± 0.01	1.27 ± 0.01	88.87 ± 0.06	−1.73 ± 0.08	26.15 ± 0.16	−0.07 ± 0.00
Maria Anna	14.07 ± 0.16	9.97 ± 0.02	4.69 ± 0.01	1.41 ± 0.01	71.43 ± 0.17	5.33 ± 0.09	25.67 ± 0.06	0.05 ± 0.00
Maria Linda	14.23 ± 0.21	6.95 ± 0.03	3.56 ± 0.03	2.04 ± 0.03	58.71 ± 0.10	1.85 ± 0.07	27.74 ± 0.11	0.07 ± 0.01
Silver Giant	12.21 ± 0.20	11.33 ± 0.12	3.53 ± 0.02	1.08 ± 0.02	51.07 ± 0.08	0.62 ± 0.08	25.46 ± 0.10	0.02 ± 0.00
Silver Ray	11.96 ± 0.10	14.51 ± 0.05	3.69 ± 0.01	0.82 ± 0.01	49.48 ± 0.14	2.95 ± 0.15	21.86 ± 0.15	0.13 ± 0.00
Silver Star	11.95 ± 0.12	15.15 ± 0.05	3.68 ± 0.02	0.79 ± 0.01	48.62 ± 0.17	2.79 ± 0.12	28.21 ± 0.09	0.10 ± 0.00

The data are presented as the mean (*n* = 3) ± S.D.

**Table 3:** Descriptive statistics for the variables of sucrose, glucose, fructose, and total sugars (g kg^−1^ FW) of the 84 cultivars studied. For each compound, minimum, maximum, and mean values and standard deviation (SD) are reported.

Sugar	Min	Max	Mean	SD
Sucrose	48.9	87.38	67.22	8.71
Glucose	4.15	12.49	8.06	2.09
Fructose	5.47	13.37	8.79	1.71
Sorbitol	0.70	4.44	1.90	0.78
Total sugars	60.11	115.29	85.96	11.07

**Table 4:** Content and distribution of sugars (g kg^−1^ FW) in yellow and white peaches and in yellow and white nectarines.

Sugar	Yellow Peaches	White Peaches	Yellow Nectarines	White Nectarines
Sucrose	64.52 ± 9.05	68.42 ± 5.35	69.98 ± 8.32	59.94 ± 5.11
Glucose	7.73 ± 2.58	10.30 ± 1.73	8.21 ± 1.72	6.61 ± 0.61
Fructose	8.46 ± 1.88	9.53 ± 1.18	8.99 ± 1.71	8.17 ± 1.28
Sorbitol	1.61 ± 1.88	0.95 ± 0.13	2.20 ± 0.89	1.91 ± 0.40
Glucose/Fructose	0.91	1.08	0.91	0.80
Total Sugars	82.33 ± 11.64	89.20 ± 7.19	89.38 ± 10.46	76.62 ± 5.52

The data are presented as the mean (*n* = 3) ± S.D.

**Table 5:** Two-way ANOVA of sugar contents for type of fruit (peach vs. nectarine) and color (white vs. yellow) of peach fruits. F statistic and *p*-value of each factor and their interaction are reported for each variable.

	Sugar								Total Sugars	
	Sucrose		Glucose		Fructose		Sorbitol			
Factor	F	*p*-Value	F	*p*-Value	F	*p*-Value	F	*p*-Value	F	*p*-Value
Type	0.39	0.534	7.50	0.008	0.667	0.413	13.85	<0.001	0.810	0.371
Color	1.61	0.209	0.70	0.408	0.058	0.811	5.26	0.024	0.915	0.342
Interaction	8.24	0.005	12.71	<0.001	3.469	0.065	0.803	0.373	10.2	0.002

**Table 6:** Total Phenolic Contents (TPC), Total Carotenoid Contents (TCC) and Antioxidant Activity (DPPH) evaluated in yellow and white flesh of nectarine and peaches cultivars.

Yellow-Flesh Nectarines	TPC mg GAE g^−1^	TCC mg g^−1^	DPPH I_50_ (mg mL^−1^)	Yellow-Flesh Nectarines	TPC mg GAE g^−1^	TCC mg g^−1^	DPPH I_50_ (mg mL^−1^)
Alitop	0.922 ± 0.24	5.257 ± 0.19	42.94 ± 0.49	Maria Dolce	0.552 ± 0.27	3.587 ± 0.37	24.25 ± 0.59
Alma	0.654 ± 0.16	4.922 ± 0.21	28.05 ± 0. 39	Maria Dorata	0.734 ± 0.33	8.764 ± 0.25	28.86 ± 0.53
Amiga	0.347 ± 0.22	9.629 ± 0.34	26.13 ± 0.64	Maria Laura	0.253 ± 0.26	11.003 ± 0.29	50.04 ± 0.72
Antares	0.650 ± 0.33	6.003 ± 0.35	32.31 ± 0.45	Max	1.139 ± 0.25	7.353 ± 0.33	13.99 ± 0.64
August Red	0.466 ± 0.28	8.554 ± 0.45	16.7 ± 0.77	Morsiani 51	0.740 ± 0.31	4.585 ± 0.22	22.87 ± 0.81
Big Top	1.645 ± 0.25	10.118 ± 0.46	40.92 ± 0.45	Morsiani 60	0.727 ± 0.24	5.978 ± 0.30	29.17 ± 0.55
Claudia	0.557 ± 0.33	6.048 ± 0.39	31.35 ± 0.59	Nectaross	0.767 ± 0.29	10.001 ± 0.24	18.90 ± 0.64
Diamond Princess	0.318 ± 0.26	5.093 ± 038	42.54 ± 0.43	Orion	0.248 ± 0.24	11.775 ± 0.25	15.01 ± 0.73
Diamond Ray	0.525 ± 0.31	7.921 ± 0.0.33	37.05 ± 0.47	Red Jewel	0.364 ± 0.28	9.931 ± 0.31	26.06 ± 0.82
Fire Top	0.836 ± 0.24	5.107 ± 0.32	33.44 ± 0.52	Silvana	0.363 ± 0.30	6.931 ± 0.28	144.65 ± 0.93
Gian Laura Dolce	1.025 ± 0.26	8.601 ± 0.27	32.20 ± 0.45	Spring Red	1.213 ± 0.29	9.701 ± 0.26	67.17 ± 0.55
Gioia	0.538 ± 0.31	6.581 ± 0.34	89.85 ± 0.69	Star Bright	0.419 ± 0.26	3.029 ± 0.29	66.53 ± 0.62
Guerriera	0.900 ± 0.25	10.856 ± 0.27	40.16 ± 0.61	Stark Redgold	1.201 ± 0.33	8.938 ± 0.35	13.70 ± 0.51
Honey Kist	0.869 ± 0.32	11.012 ± 0.32	69.94 ± 0.57	Summer Grand	0.868 ± 0.25	5.903 ± 0.27	31.33 ± 0.78
Honey Royale	0.396 ± 0.27	6.764 ± 0.46	29.14 ± 0.72	Super Super Star	1.130 ± 0.31	4.904 ± 0.31	30.77 ± 0.62
Independence	0.322 ± 0.25	6.422 ± 0.38	62.60 ± 0.62	Sweet Lady	0.311 ± 0.27	5.458 ± 0.28	13.55 ± 0.76
Lady Erika	0.491 ± 0.33	5.26 ± 0.28	17.92 ± 0.65	Sweet Red	1.047 ± 0.34	4.887 ± 0.33	12.32 ± 0.60
Lady Star	0.477 ± 0.28	5.851 ± 0.32	16.85 ± 0.52	Vega	0.660 ± 0.28	16.652 ± 0.30	19.57 ± 0.82
Licinia	0.850 ± 0.29	12.383 ± 0.36	34.80 ± 0.56	Venus	0.366 ± 0.25	3.840 ± 0.24	19.46 ± 0.72
Maeba Top	0.111 ± 0.26	5.388 ± 0.29	93.57 ± 0.41	Weinberger	0.265 ± 0.30	4.717 ± 0.27	56.26 ± 0.79
Maria Aurelia	0.522 ± 0.29	7.694 ± 0.34	37.83 ± 0.55				
Maria Camilla	0.218 ± 0.30	4.771 ± 0.28	51.79 ± 0.75				
Maria Carla	0.689 ± 0.35	3.831 ± 0.26	26.74 ± 0.80				
**White-flesh** **nectarines**	**TPC** **mg GAE g^−1^**	**TCC** **mg g^−1^**	**DPPH** **I_50_ (mg mL^−1^)**				
Caldesi 2000	0.557 ± 0.31	2.682 ± 0.22	55.12 ± 0.55				
Caldesi 2010	0.514 ± 0.26	2.255 ± 0.30	25.03 ± 0.52				
Caldesi 2020	0.929 ± 0.30	2.876 ± 0.26	23.11 ± 0.65				
Maria Anna	0.608 ± 0.26	6.707 ± 0.33	19.39 ± 0.63				
Maria Linda	0.192 ± 0.29	3.381 ± 0.29	35.46 ± 0.49				
Silver Giant	0.275 ± 0.33	2.423 ± 0.25	37.28 ± 0.53				
Silver Ray	0.728 ± 028	3.946 ± 0.30	38.79 ± 1.25				
Silver Star	0.364 ± 0.31	3.207 ± 0.29	35.61 ± 0.63				
**Yellow-flesh peaches**	**TPC** **mg GAE g^−1^**	**TCC** **mg g^−1^**	**DPPH** **I_50_ (mg mL^−1^)**	**Yellow-flesh peaches**	**TPC** **mg GAE g^−1^**	**TCC** **mg g^−1^**	**DPPH** **I_50_ (mg mL^−1^)**
Elegant Lady	0.552 ± 0.17	5.220 ± 0.24	26.79 ± 0.51	Rich Lady	0.739 ± 0.13	17.816 ± 0.27	117.72 ± 1.05
Fayette	0.429 ± 0.15	7.786 ± 0.33	63.80 ± 0.55	Rome Star	0.860 ± 0.17	4.528 ± 0.25	27.58 ± 1.23
Flavorcrest	1.164 ± 0.18	5.712 ± 0.29	120.26 ± 1.13	Summer Rich	0.321 ± 0.20	5.811 ± 0.31	39.80 ± 0.48
Glohaven	0.585 ± 0.20	9.935 ± 0.31	41.64 ± 0.88	Suncrest	0.387 ± 0.16	4.776 ± 0.34	27.31 ± 0.54
Grenat	0.408 ± 0.14	6.744 ± 0.23	20.79 ± 0.77	Symphonie	0.434 ± 0.12	4.252 ± 0.29	14.47 ± 0.40
Guglielmina	0.660 ± 0.16	3.680 ± 0.25	22.99 ± 0.67	Vistarich	1.951 ± 0.16	12.314 ± 0.33	27.15 ± 0.38
Kaweah	0.780 ± 0.13	5.250 ± 0.21	49.90 ± 0.49	Zee Lady	0.856 ± 0.20	8.482 ± 0.27	13.47 ± 0.46
Lara Star	0.279 ± 0.21	6.874 ± 0.32	93.48 ± 0.52	Babygold 7	0.693 ± 0.19	5.385 ± 0.22	38.29 ± 1.08
Lizbeth	0.387 ± 0.14	4.348 ± 0.27	84.85 ± 0.51	Babygold 9	0.434 ± 0.15	9.624 ± 0.31	25.64 ± 0.66
Maria Marta	0.832 ± 0.16	9.068 ± 0.25	22.84 ± 0.73	Carson	0.557 ± 0.22	5.309 ± 0.34	55.37 ± 0.51
Maria Silvia	0.272 ± 0.18	10.164 ± 0.28	18.04 ± 0.49	Cotogna Poggio	0.776 ± 0.19	6.816 ± 0.28	37.97 ± 0.76
Padana	0.250 ± 0.11	4.991 ± 0.30	35.95 ± 0.51				
Red Coast	0.562 ± 0.17	4.705 ± 0.31	51.99 ± 0.41				
Redhaven	0.495 ± 0.15	5.633 ± 0.29	17.46 ± 0.43				
Red Valley	0.217 ± 0.17	5.591 ± 0.24	34.30 ± 0.42				
**White-flesh** **peaches**	**TPC** **mg GAE g^−1^**	**TCC** **mg g^−1^**	**DPPH** **I_50_ (mg mL^−1^)**				
Greta	0.393 ± 0.21	2.180 ± 0.24	29.04 ± 0.38				
Maria Bianca	1.007 ± 0.16	8.221 ± 0.31	64.26 ± 0.40				
Maria Regina	0.468 ± 0.22	2.908 ± 0.27	31.94 ± 0.37				
Michelini	0.581 ± 0.18	1.319 ± 0.36	22.67 ± 0.48				
Rosa del West	1.377 ± 0.24	7.751 ± 0.31	54.06 ± 0.50				
Tardivo Zuliani	0.328 ± 0.21	1.225 ± 0.29	36.24 ± 0.45				

The data are presented as the mean (*n* = 3) ± S.D.

## Supplementary Materials

The following supporting information can be downloaded at https://www.mdpi.com/article/10.3390/plants12081618/s1: Table S1: Peaches and nectarines cultivars used in this study; Table S2: Two-way ANOVA for all variables analyzing two-levels factors: Type: Peach or Nectarine Color: Yellow or White; Table S3: Sugar content (g kg^−1^ FW) in fruit of yellow and white peach cultivars; Table S4: Sugar content (g kg^−1^ FW) in fruit of yellow and white nectarine cultivars; Table S5: Concentration (mg/kg FW) of phenolic compounds in yellow-and white-flesh nectarines (nd: not determined); Table S6: Concentration (mg/kg FW) of phenolic compound in yellow-and white-flesh peaches (nd: not determined).
