# Supplementary material for: Evaluation of the Fruit Quality and Phytochemical Compounds in Peach and Nectarine Cultivars
Source: Plants (Basel). 2023 Apr 12;12(8):1618. doi: 10.3390/plants12081618 (PMC10144225; doi:10.3390/plants12081618)
Supplement: Supplementary file 1 [file plants-12-01618-s001.zip › Table S2.pdf]

Table S2. Two-way ANOVA for all variables analyzing two-levels factors:
Type: Peach or Nectarine **Color**: Yellow or White

**⁰Brix** (%) (Significant F at 5% level are marked in red)					
Cases	Sum of Squares	df	Mean Square	F	p
Color	0.593	1	0.593	0.287	0.594
Type	2.377	1	2.377	1.149	0.287
Color ✻ Type	0.356	1	0.356	0.172	0.680
Residuals	165.6	80	2.096		**a**

**Total Acidity** (g malic acid L^{-1})(Significant F at 5% level are marked in red)					
Cases	Sum of Squares	df	Mean Square	F	p
Color	0.753	1	0.753	0.140	0.710
Type	25.22	1	25.22	4.676	0.034
Color ✻ Type	5.818	1	5.818	1.079	0.302
Residuals	432.0	80	5.394		**b**

**pH** (Significant F at 5% level are marked in red)					
Cases	Sum of Squares	df	Mean Square	F	p
Color	0.182	1	0.182	1.772	0.187
Type	0.008	1	0.008	0.079	0.780
Color ✻ Type	0.140	1	0.140	1.368	0.246
Residuals	8.211	80	0.103		**c**

**RI index** (Significant F at 5% level are marked in red)					
Cases	Sum of Squares	df	Mean Square	F	p
Color	0.040	1	0.040	0.324	0.571
Type	0.135	1	0.135	1.094	0.299
Color ✻ Type	0.047	1	0.047	0.379	0.540
Residuals	9.868	80	0.123		**d**

L (Significant F at 5% level are marked in red)					
Cases	Sum of Squares	df	Mean Square	F	p
Color	731.4	1	731.4	6.690	0.012
Type	2168	1	2168	19.827	< 0.001
Color ✻ Type	1757	1	1757	16.076	< 0.001
Residuals	8746	80	109.3		**e**

Polyphenols (mg GAE g^{-1}) (Significant F at 5% level are marked in red)					
Cases	Sum of Squares	df	Mean Square	F	p
Color	0.002	1	0.002	0.020	0.888
Type	0.065	1	0.065	0.562	0.456
Color ✻ Type	0.106	1	0.106	0.915	0.342
Residuals	9.226	80	0.115		**f**

DPPH (Significant F at 5% level are marked in red)					
Cases	Sum of Squares	df	Mean Square	F	p
Color	186.3	1	186.3	0.276	0.601
Type	365.5	1	365.5	0.542	0.464
Color ✻ Type	1.017	1	1.017	0.002	0.969
Residuals	5.395e+4	80	674.4		**g**

Chlorogenic acid (Significant F at 5% level are marked in red)					
Cases	Sum of Squares	df	Mean Square	F	p
Color	1795	1	1795	1.251	0.267
Type	7294	1	7294	5.085	0.027
Color ✻ Type	3899	1	3899	2.718	0.103
Residuals	1.148e+5	80	1435		**h**

**Neochlorogenic acid** (Significant F at 5% level are marked in red)					
Cases	Sum of Squares	df	Mean Square	F	p
Color	405.3	1	405.3	0.741	0.392
Type	3519	1	3519	6.437	0.013
Color ✻ Type	737.1	1	737.1	1.348	0.249
Residuals	4.734e+4	80	546.7		**i**

**Catechin** (Significant F at 5% level are marked in red)					
Cases	Sum of Squares	df	Mean Square	F	p
Color	2820	1	2820	1.614	0.208
Type	21.13	1	21.13	0.012	0.913
Color ✻ Type	1045	1	1045	0.598	0.442
Residuals	13.98e+5	80	1747		**l**

**Epicatechin** (Significant F at 5% level are marked in red)					
Cases	Sum of Squares	df	Mean Square	F	p
Color	169.8	1	169.8	1.259	0.265
Type	139.0	1	139.0	1.031	0.313
Color ✻ Type	107.4	1	107.4	0.797	0.375
Residuals	1.079e+4	80	134.9		**m**
