# Supplementary material for: Evaluation of the Fruit Quality and Phytochemical Compounds in Peach and Nectarine Cultivars
Source: Plants (Basel). 2023 Apr 12;12(8):1618. doi: 10.3390/plants12081618 (PMC10144225; doi:10.3390/plants12081618)
Supplement: Supplementary file 1 [file plants-12-01618-s001.zip › Table S3.pdf]

Table S3. Sugar content (g kg^{-1} FW) in fruits of yellow and white peach cultivars.

**Yellow-flesh cvs**	**Sucrose**	**Glucose**	**Fructose**	**Sorbitol**	**Total sugars**
Elegant Lady	58.79±1.3	6.96±0.2	9.79±0.6	2.23±0.09	77.77±1.07
Fayette	74.12±0.7	12.14±0.4	10.08±0.1	1.82±0.06	98.16±0.42
Flavorcrest	73.37±1.0	9.15±0.6	9.97±0.2	1.82±0.05	94.30±1.02
Glohaven	60.05±0.6	4.57±0.3	6.20±0.2	1.56±0.09	72.38±0.64
Grenat	81.99±0.9	10.05±0.2	7.48±0.4	1.77±0.05	101.30±1.35
Guglielmina	48.96±0.5	4.14±0.1	5.47±0.2	1.52±0.2	60.11±0.62
Kaweah	53.65±0.4	6.11±0.2	8.06±0.2	1.80±0.3	69.62±0.67
Lara Star	76.06±0.5	10.95±0.05	9.02±0.09	1.83±0.09	97.86±0.33
Lizbeth	80.23±0.8	6.11±0.2	9.91±0.2	2.01±0.2	99.66±0.79
Maria Marta	70.03±0.3	11.3±0.07	7.64±0.3	2.16±0.04	91.17±0.66
Maria Silvia	68.31±0.8	10.40±0.4	10.32±0.4	1.78±0.3	90.81±1.60
Padana	66.91±0.4	7.01±0.1	9.04±0.06	1.68±0.04	84.64±0.55
Red Coast	58.31±0.4	7.41±0.09	6.44±0.2	1.13±0.07	73.30±0.10
Redhaven	54.71±0.5	6.15±0.1	7.59±0.2	1.96±0.09	70.41±0.28
Red Valley	59.92±0.4	6.22±0.1	8.33±0.1	1.46±0.08	75.93±0.28
Rich Lady	66.21±0.5	5.21±0.1	6.60±0.2	1.22±0.04	79.23±0.33
Rome Star	63.90±1.1	10.94±0.07	11.53±0.2	2.32±0.4	88.47±1.44
Summer Rich	50.21±0.4	10.76±0.08	9.91±0.4	1.39±0.06	72.27±0.76
Suncrest	58.26±0.3	5.54±0.2	9.85±0.3	2.19±0.1	75.84±0.61
Symphonie	75.89±0.3	12.49±0.1	12.56±0.1	1.67±0.07	102.61±0.44
Vistarich	51.65±0.5	6.93±0.2	8.47±0.2	1.04±0.05	68.08±0.18
Zee Lady	62.51±0.4	7.69±0.1	8.83±0.06	1.24±0.07	81.41±0.11
Babygold 7	64.33±0.1	6.73±0.07	4.92±0.08	1.16±0.06	77.14±0.38
Babygold 9	69.32±0.2	7.44±0.2	5.64±0.09	1.35±0.07	83.73±0.28
Carson	63.83±0.2	5.57±0.09	4.95±0.07	1.10±0.04	75.32±0.40
Cotogna del Poggio	66.14±0.1	5.99±0.1	5.73±0.10	1.09±0.03	78.96±0.19
**White-flesh cvs**					
Greta	68.72±0.2	11.14±0.1	8.69±0.2	1.03±0.05	89.59±0.60
Maria Bianca	69.72±0.8	12.09±0.1	11.3±0.2	0.70±0.04	93.86±0.47
Maria Regina	63.83±0.2	10.30±0.1	9.96±0.1	0.92±0.05	85.02±0.40
Michelini	76.08±0.1	11.48±0.2	10.2±0.3	1.01±0.04	89.99±0.41
Rosa del West	71.12±0.3	9.51±0.2	8.34±0.1	1.05±0.08	89.99±0.39
Tardivo Zuliani	61.05±0.2	7.29±0.1	8.59±0.1	1.02±0.04	77.96±0.50

The data are presented as the mean ± S.D.
