# Supplementary material for: Evaluation of the Fruit Quality and Phytochemical Compounds in Peach and Nectarine Cultivars
Source: Plants (Basel). 2023 Apr 12;12(8):1618. doi: 10.3390/plants12081618 (PMC10144225; doi:10.3390/plants12081618)
Supplement: Supplementary file 1 [file plants-12-01618-s001.zip › Table S4.pdf]

Table S4. Sugar content (g kg^{-1} FW) in fruits of yellow and white nectarine cultivars

Yellow-flesh cvs	Sucrose	Glucose	Fructose	Sorbitol	Total sugars
Alitop	85.44±0.22	10.11±0.09	11.23±0.14	1.12±0.04	107.82±0.39
Alma	72.21±0.13	17.04±0.10	11.37±0.79	1.39±0.07	92.02±1.39
Amiga	66.44±0.19	9.44±0.10	10.50±0.22	3.32±0.11	89.70±0.15
Antares	76.67±0.14	9.20±0.35	10.95±0.10	3.29±0.05	100.11±0.33
August Red	84.89±0.10	8.15±0.06	10.19±0.14	3.24±0.08	106.48±0.17
Big Top	87.38±0.16	11.24±0.06	13.37±0.11	3.30±0.14	81.97±1.04
Claudia	57.92±0.11	6.32±0.10	8.61±0.16	2.60±0.06	75.45±0. 36
Diamond Princess	65.01±0.30	7.52±0.14	7.94±0.10	2.06±0.09	82.53±0.28
Diamond Ray	76.06±0.16	8.90±0.11	9.02±0.09	2.29±0.04	96.27±0.10
Fire Top	79.06±0.42	11.66±0.11	12.25±0.11	4.44±0.11	107.41±0.50
Gianna Laura Dolce	52.28±0.17	7.76±0.14	8.54±0.10	0.96±0.06	69.03±0.10
Gioia	64.27±0.13	7.67±0.11	8.24±0.11	2.21±0.10	82.48±0.37
Guerriera	67.53±0.28	6.39±0.14	6.99±0.09	1.98±0.04	82.91±0.41
Honey Kist	70.09±0.31	8.60±0.09	7.59±0.19	2.07±0.03	88.36±0.23
Honey Royale	66.15±0.29	5.93±0.17	6.76±0.17	1.87±0.08	80.67±0.13
Indipendence	58.41±0.22	4.21±0.17	8.27±0.07	1.23±0.09	72.11±0.15
Lady Erika	69.72±0.28	5.94±0.10	8.66±0.10	1.05±0.07	85.37±0.61
Lady Star	66.44±0.13	8.77±0.05	8.16±0.09	1.15±0.06	84.52±0.09
Licinia	56.09±0.11	8.78±0.13	10.01±0.13	2.94±0.09	77.82±0.19
Maeba Top	70.14±0.25	9.98±0.11	9.04±0.09	2.38±0.15	91.54±0.17
Maria Aurelia	66.72±0.12	8.59±0.04	10.55±0.10	2.16±0.06	88.02±0.29
Maria Camilla	68.28±0.32	7.91±0.10	10.04±0.09	1.65±0.09	87.89±0.24
Maria Carla	69.95±0.36	7.72±0.12	9.86±0.13	2.24±0.07	89.78±0.56
Maria Dolce	64.29±0.26	7.52±0.12	8.76±0.11	1.34±0.27	81.91±0.45
Maria Dorata	61.70±0.14	5.31±0.28	6.13±0.08	0.98±0.04	74.12±0.10
Maria Laura	64.98±0.19	4.56±0.10	6.09±0.12	1.08±0.10	76.70±0.17
Max	71.05±0.17	9.53±0.12	8.32±0.10	2.79±0.06	91.69±0.18
Morsiani 51	68.05±0.21	8.89±0.11	7.31±0.14	1.12±0.04	85.45±0.22
Morsiani 60	62.09±0.22	8.15±0.24	8.61±0.14	1.79±0.06	80.63±0.10
Nectaross	82.27±0.15	10.13±0.06	8.66±0.06	2.01±0.06	103.07±0.20
Orion	74.18±0.22	10.04±0.19	7.95±0.09	1.98±0.05	94.16±0.44
Red Jewel	80.02±0.17	9.98±0.09	8.76±0.06	2.22±0.04	100.92±0.29
Silvana	65.93±0.11	8.20±0.11	9.68±0.08	2.11±0.05	85.91±0.18
Spring Bright	77.27±0.23	10.03±0.05	8.79±0.05	3.02±0.04	99.11±0.18
Spring Red	63.90±0.26	6.92±0.23	8.16±0.05	2.19±0.05	81.18±0.17
Star Bright	87.27±0.32	6.87±0.06	7.24±0.11	2.34±0.09	103.72±0.20
Stark Redgold	74.37±0.15	7.43±0.06	8.57±0.04	2.11±0.04	92.456±0.13
Summer Grand	76.29±0.21	9.78±0.08	10.86±0.13	1.34±0.04	98.27±0.34
Superior Super Star	61.06±0.25	7.07±0.08	8.47±0.10	1.75±0.11	78.35±0.58
Sweet Lady	77.29±0.18	8.39±0.15	5.72±0.08	3.94±0.10	95.34±0.20
Sweet Red	71.24±0.14	8.78±0.17	9.91±0.26	4.41±0.21	94.34±0.17
Vega	70.69±0.16	6.28±0.05	7.27±0.08	3.34±0.12	88.12±0.72
Venus	62.57±0.32	8.00±0.18	9.45±0.10	2.26±0.10	1145.29±0.23

Weinberger	65.84±0.24	11.47±0.17	12.64±0.09	1.69±0.04	91.64±0.53
**White-flesh cvs**					
Caldesi 2000	66.48±0.1	11.37±0.3	6.20±0.07	1.39±0.07	84.71±0.19
Caldesi 2010	58.94±0.08	10.50±0.2	7.09±0.1	3.32±0.1	76.73±0.21
Caldesi 2020	57.94±0.2	10.19±0.05	7.39±0.2	3.24±0.08	75.23±0.27
Maria Anna	58.52±0.1	11.23±0.1	6.87±0.04	1.12±0.04	77.93±0.19
Maria Linda	55.35±0.1	10.95±0.09	5.61±0.08	3.29±0.05	70.56±0.26
Silver Giant	60.37±0.03	9.45±0.1	5.93±0.07	2.26±0.08	75.68±0.82
Silver Ray	68.31±0.1	9.02±0.09	6.90±0.1	2.29±0.04	83.85±0.39
Silver Star	53.48±0.2	8.61±0.2	7.01±0.2	2.60±0.06	68.79±0.42

The data are presented as the mean ± S.D.
