# Supplementary material for: Evaluation of the Fruit Quality and Phytochemical Compounds in Peach and Nectarine Cultivars
Source: Plants (Basel). 2023 Apr 12;12(8):1618. doi: 10.3390/plants12081618 (PMC10144225; doi:10.3390/plants12081618)
Supplement: Supplementary file 1 [file plants-12-01618-s001.zip › Table S5.pdf]

Table S5. Concentration (mg/Kg FW) of phenolic compound in yellow-and white-flesh nectarines (nd: not determined)

	Neochlorogenic acid	Chlorogenic acid	Cathechin	Epicathechin	Total Flavan-3-ols	Total HYdroxycinnamic acids
**Yellow-flesh cvs**						
Alitop	10.60	12.97	nd	3.92	3.92	23.57
Alma	68.72	121.02	69.67	22.52	92.19	189.74
Amiga	38.99	52.12	nd	nd	-	91.10
Antares	26.53	65.82	122.21	nd	122.21	92.35
August Red	24.51	54.66	61.25	18.79	80.04	79.17
Big Top	20.96	22.88	nd	22.52	22.52	43.85
Claudia	18.20	17.80	nd	30.33	30.33	36.00
Diamond Princess	21. 49		26.78	nd	26.78	59.54
Diamond Ray	69.85	77.29	27.63	3.66	31.29	147.14
Fire Top	15.11	25.55	11.16	28.34	39.51	40.66
G. Laura Dolce	28.38	38.68	31.75	38.01	67.06	67.06
Gioia	6.20	10.28	nd	26.21	26.21	16.49
Guerriera	9.33		nd	5.20	5.20	9.33
Honey Kist	8.24	4.16	nd	42.40	42.40	12.41
Honey Royale	47.27	46.55	nd	21.53	21.53	93.82
Indipendence	20.99	37.09	nd	25.79	25.79	58.08
Lady Erika	61.43	61.69	59.58	27.49	87.08	123.10
Lady Star	46.96	40.84	132.57	12.58	145.15	87.79
Licinia	19.57	29.92	15.85	33.88	49.73	49.49
Maeba Top	9.83	7.81	nd	20.39	20.39	17.64
Maria Aurelia	16.40	15.52	nd	11.87	11.87	31.92
Maria Camilla	21.47	12.23	nd	27.35	27.35	33.70
Maria Carla	32.39	60.61	30.47	25.08	55.55	93.00
Maria Dolce	66.16	19.31	86.99	35.73	122.72	85.46
Maria Dorata	49.22	73.07	15.42	26.64	42.06	117.58
Maria Laura	31.28	39.16		16.84	16.84	70.44

Max	128.27	125.27	77.62	19.68	97.30	253.54
Morsiani 51	72.01	158.63	72.51	33.88	106.39	230.64
Morsiani 60	25.40	40.36	50.78	nd	50.78	65.76
Nectaross	49.16	84.61	68.81	23.80	92.61	133.78
Orion	58.21	87.04	125.47	22.24	147.71	145.25
Red Jewel	41.48	50.77	nd	nd	-	92.26
Silvana	5.82	4.88	nd	nd	-	10.70
Spring Bright	20.31	8.29	nd	16.13	16.13	28.61
Spring Red	37.62	23.70	nd	nd	nd	61.32
Star Bright	20.32	94.33	28.63	nd	28.63	139.68
Stark Redgold	55.72	113.34	117.80	18.55	136.35	169.06
Summer Grand	33.97	37.31	25.50	24.51	50.01	71.28
Sup Super Star	32.05	25.57	83.72	20.81	104.54	57.62
Sweet Lady	66.42	52.96	41.55	23.23	64.78	119.38
Sweet Red	12.16	17.56	10.88	23.80	34.68	29.71
Vega	47.03	47.94	21.373.9	35.73	57.11	94.97
Venus	27.56	34.84	nd	23.66	76.00	62.40
Weinberger	8.77	15.32	nd	37.29	37.29	24.02
**White flesh cvs**						
Caldesi 2000	31.38	50.51	nd	15.56	15.56	81.89
Caldesi 2010	22.24	39.01	20.96	12.30	33.26	61.25
Caldesi 2020	25.62	42.66	16.55	14.78	31.33	68.28
Maria Anna	50.29	50.75	113.83	20.39	134.22	101.04
Maria Linda	48.31	45.62	88.75	17.41	106.16	93.93
Silver Giant	78.23	162.06	61.15	21.24	82.39	240.29
Silver Ray	30.50	38.80	68.96	34.02	102.98	69.29
Silver Star	108.08	184.67	99.77	12.58	193.09	292.75
