# Supplementary material for: Evaluation of the Fruit Quality and Phytochemical Compounds in Peach and Nectarine Cultivars
Source: Plants (Basel). 2023 Apr 12;12(8):1618. doi: 10.3390/plants12081618 (PMC10144225; doi:10.3390/plants12081618)
Supplement: Supplementary file 1 [file plants-12-01618-s001.zip › Table S6.pdf]

Table S6. Concentration (mg/Kg FW) of phenolic compound in yellow-and white-flesh peaches (nd: not determined)

	Neochlorogenic acid	Chlorogenic acid	Cathechin	Epicathechin	Total Flavan-3-ols	Total Hydroxycinnamic acids
**Yellow-flesh cvs**						
Elegant Lady	32.96	59.24	47.09	34.17	81.25	92.21
Fayette	12.47	16.69	nd	20.53	20.53	29.16
Flavorcrest	2.92	7.40	nd	30.76	30.76	10.32
Glohaven	10.60	12.85	nd	nd	-	23.45
Grenat	1057	15.13	21.34	25.64	47.03	25.70
Guglielmina	19.65	26.69	27.29	33.47	60.76	46.34
Kaweah	7.62	6.13	18.26	36.15	54.41	13.75
Lara Star	6.28	9.01	nd	19.97	19.97	15.29
Lizbeth	6.25	16.02	nd	24.08	24.08	22.27
Maria Marta	50.58	51.47	120.64	17.27	137.91	102.05
Maria Silvia	9.35	7.60	27.35	36.01	63.36	16.94
Padana	25.98	23.80	nd	21.67	21.67	49.78
Red Coast	46.04	26.51	65.55	17.41	82.96	72.55
Redhaven	14.12	11.12	15.99	24.23	40.22	25.25
Red Valley	24.90	57.49	58.45	18.83	77.28	77.28
Rich Lady	44.44	98.94	71.37	28.91	100.28	143.37
Rome Star	27.37	41.15	77.33	18.97	96.31	68.52
Summer Rich	23.58	116.22	102.61	36.15	138.76	139.80
Suncrest	40.98	46.09	38.00	19.68	57.68	87.07
Symphonie	37.28	50.10	68.25	25.93	94.17	87.38
Vistarich	8.70	15.32	nd	37.29	37.29	24.02
Zee Lady	24.95	55.58	132.71	23.94	156.66	80.50
Babygold 7	20.84	21.78	36.58	41.83	78.41	42.62
Babygold 9	40.98	40.24	34.45	30.33	64.78	81.22
Carson	18.04	29.56	nd	nd	-	47.60
Cotogna Poggio	101.55	146.98	118.51	50.35	168.87	248.53
**White flesh cvs**						

Greta	46.67	63.64	77.34	21.53	98.87	110.30
Maria Bianca	11.48	20.89	nd	nd	-	32.38
Maria Regina	11.01	16.45	nd	34.88	34.88	27.46
Michelini	44.41	31.28	92.10	15.42	107.52	75.70
Rosa del West	21.30	53.53	117.24	23.09	140.33	74.83
Tardivo Zuliani	7.02	11.29	nd	18.83	18.83	18.31
